# Concomitant nevirapine impacts pharmacokinetic exposure to the antimalarial artemether-lumefantrine in African children

**DOI:** 10.1371/journal.pone.0186589

**Published:** 2017-10-24

**Authors:** Liusheng Huang, Vincent Carey, Jane C. Lindsey, Florence Marzan, David Gingrich, Bobbie Graham, Linda Barlow-Mosha, Phionah K. Ssemambo, Portia Kamthunzi, Sharon Nachman, Sunil Parikh, Francesca T. Aweeka, the IMPAACT P1079 protocol team

**Affiliations:** 1 Drug Research Unit, Department of Clinical Pharmacy, University of California, San Francisco, CA, United States of America; 2 Center for Biostatistics in AIDS Research, Harvard TH Chan School of Public Health, Boston, MA, United States of America; 3 Frontier Science and Technology Research Foundation, Buffalo, NY, United States of America; 4 Makerere University, MUJHU Research Collaboration, Kampala, Uganda; 5 Kamuzu Central Hospital, Private Bag A-104, Lilongwe, Malawi; 6 School of Medicine, Stony Brook University, Stony Brook, NY, United States of America; 7 Yale School of Public Health, New Haven, CT, United States of America; University of New South Wales, AUSTRALIA

## Abstract

**Background:**

The antiretroviral drug nevirapine and the antimalarial artemisinin-based combination therapy artemether-lumefantrine are commonly co-administered to treat malaria in the context of HIV. Nevirapine is a known inhibitor of cytochrome P450 3A4, which metabolizes artemether and lumefantrine. To address the concern that the antiretroviral nevirapine impacts the antimalarial artemether-lumefantrine pharmacokinetics, a prospective non-randomized controlled study in children presenting with uncomplicated malaria and HIV in sub-Saharan Africa was carried out.

**Methods:**

Participants received artemether-lumefantrine (20/120 mg weight-based BID) for 3 days during nevirapine-based antiretroviral therapy (ART) co-administration (158–266 mg/m^2^ QD). HIV positive participants who were not yet on ART drugs were also enrolled as the control group. The target enrollment was children aged 3–12 years (n = 24 in each group). Intensive pharmacokinetics after the last artemether-lumefantrine dose was assessed for artemether, its active metabolite dihydroartemisinin, and lumefantrine. Pharmacokinetic parameters (area under the plasma concentration vs. time curve (AUC), maximum concentration and day 7 lumefantrine concentrations) were estimated using non-compartmental methods and compared to controls.

**Results:**

Nineteen children (16 on nevirapine and three not on ART) enrolled. Fifteen of the 16 (aged 4 to 11 years) on nevirapine-based ART were included in the pharmacokinetic analysis. Due to evolving WHO HIV treatment guidelines, insufficient children were enrolled in the control group (n = 3), so the pharmacokinetic data were compared to a historical control group of 20 HIV-uninfected children 5–12 years of age who also presented with malaria and underwent identical study procedures. Decreases of pharmacokinetic exposure [as estimated by AUC (AUC_0-8hr_)] were marginally significant for artemether (by -46%, p = 0.08) and dihydroartemisinin (-22%, p = 0.06) in the children on nevirapine-based ART, compared to when artemether-lumefantrine was administered alone. Similarly, peak concentration was decreased by 50% (p = 0.07) for artemether and 36% (p = 0.01) for dihydroartemisinin. In contrast, exposure to lumefantrine increased significantly in the context of nevirapine [AUC_0-120hr_:123% (p<0.001); C_day7_:116% (p<0.001), C_max_: 95% (p<0.001)].

**Conclusions:**

Nevirapine-based ART increases the exposure to lumefantrine in pre-pubescent children with a trend toward diminished artemether and dihydroartemisinin exposure. These findings contrast with other studies indicating NVP reduces or results in no change in exposure of antimalarial drugs, and may be specific to this age group (4–12 years). Considering the excellent safety profile of artemether-lumefantrine, the increase in lumefantrine is not of concern. However, the reduction in artemisinin exposure may warrant further study, and suggests that dosage adjustment of artemether-lumefantrine with nevirapine-based ART in children is likely warranted.

## Introduction

Malaria, caused by *Plasmodium* parasites and transmitted by the female *Anopheles* mosquito[1], is a life-threatening disease. The World Health Organization (WHO) estimated that in 2015, 214 million new cases of malaria led to 438,000 deaths worldwide, with 70% of these deaths in children less than 5 years of age[2]. On the other hand, there are nearly 40 million people living with HIV. Among them there are 1.8 million children < 15 years with over 50% of cases in Africa. [3]. The bulk of HIV and malaria co-infection resides in sub-Saharan African. With the availability of antiretroviral therapy (ART) for HIV and artemisinin-based combination therapy (ACT) for malaria expanded in Africa, drug-drug interactions between ART and ACT must be considered among people co-infected with HIV and malaria.

ACT is recommended by the WHO as first-line for uncomplicated *Plasmodium falciparum* malaria. Artemether-lumefantrine (AL) is a widely used ACT in Africa and is the only ACT approved in the United States[4]. For HIV positive children ≥3 years, the WHO recommended efavirenz or nevirapine (NVP)-based ART as the first-line treatment when this study was initiated[5]. Although now the efavirenz-based ART is the preferred 1^st^ line treatment and NVP-based ART is one of the alternative 1^st^ line regimens for children ≥ 3 years[6], in resource-limited settings, including sub-Saharan Africa, AL is commonly used in HIV co-infected patients treated with NVP-based ART. Studies have demonstrated that efavirenz-based ART significantly reduces the pharmacokinetic exposure to AL while lopinavir/ritonavir (LPV/r)-based ART increases the exposure to long-acting lumefantrine [7–12]. The impact of NVP-based ART has been less studied and with conflicting results [9–11, 13, 14].

International Maternal Pediatric Adolescent AIDS Clinical Trials (IMPAACT) Protocol P1079 was an international prospective pharmacokinetic (PK) study designed to investigate drug-drug interactions between AL and NVP-based ART in co-infected children aged 3–12 years. Two groups of HIV-infected children were enrolled; the first stabilized on NVP-based ART and the second not yet initiating ART. Due to expanded coverage under evolving WHO HIV treatment guidelines, few children were identified that were HIV-infected and not yet on ART, and the study closed to enrollment before reaching its accrual targets. Thus, PK results for the HIV-infected children on NVP-based ART were compared to a previously studied group of HIV-uninfected children who were evaluated using the identical treatment and PK study design, to determine the impact of NVP-based ART on AL PK disposition[15]. The primary outcomes are area under the plasma concentration vs. time curve (AUC), peak concentrations and day 7 lumefantrine (LR) concentrations.

## Methods

### P1079 study design

P1079 was an observational study. There was no randomization. Target enrollment was 24 participants on NVP-based ART for at least 4 weeks prior to enrollment (“NVP” group) and 24 who had not received ART drugs in the 4 weeks prior to enrollment (“no ART” group). The sample size was calculated to detect 30% difference in mean AUC with 80% power and a 5% two-sided Type I error, assuming a coefficient of variation (CV) of 35%. Participants were enrolled at two sites in Malawi (Lilongwe and Blantyre) and one site in Kampala, Uganda. Children were recruited based on the following inclusion criteria: (a) age 3–12 years, with HIV-1 positive results tested from two time points; (b) presenting with non-severe malaria indicated by positive smear of malaria parasites along with clinical evidence of infection (fever or history of fever in the past 24 hr); (c) receiving either NVP-based ART or no ART for at least 4 weeks prior to study entry; (d) ≤3 doses of AL (constituting up to 3 of 6 total doses) at study entry or to be treated with AL; and (e) demonstrated ability to swallow study medications and parental/legal guardian consent. Exclusion criteria included: (a) grade ≥3 hemoglobin abnormalities <7.5 g/dL, as defined by the NIH [16]; (b) severe malnutrition defined as (i) body mass index (BMI) Z-score< -3SD for children ≥5 years old or (ii) Weight-for-Height <-3SD for children <5 years old; (c) receipt of a protease inhibitor or efavirenz within 4 weeks prior to entry; (d) currently receiving an antimalarial drug other than AL; (e) use of AL for a prior episode of malaria within 6 weeks of study entry; (f) pregnancy or breastfeeding, vomiting, malignancy or known allergy to milk products; and (g) use of any following drugs within 3 weeks prior to receiving study drug: carbamazepine, clarithromycin, erythromycin (oral), ketoconazole, phenobarbital, phenytoin, rifabutin, rifampin, halofantrine, and any other medication known to inhibit or induce CYP enzymes.

Participants were evaluated at study entry (day 0), days 1, 2, 3, 4, 8, 14, 28 and 42 days. Safety evaluations included collection of complete blood count (CBC) and levels of aspartate aminotransferase (AST), alanine aminotransferase (ALT) and serum creatinine at entry, days 14, 28 and 42 and signs/symptoms at all study visits. The primary safety outcome was development of any new grade ≥3 laboratory values, signs, symptoms or diagnoses as specified in the Division of AIDS Table for Grading the Severity of Adult and Pediatric Adverse Events[16].

Participants received 6 doses of co-formulated AL (Coartem®, Novartis) as per weight-based dosing guidelines, administered as two doses of AL on day 0 and day 2 and one dose on days 1 and 3, with the 6^th^ dose given on the morning of day 3 to facilitate PK sampling during the day. Dosing guidelines were: <15kg, 1 tablet (20mg/120mg artemether/lumefantrine); 15–24.9 kg, 2 tablets; 25–34.9 kg, 3 tablets; ≥35kg, 4 tablets. All doses were to be taken with a glass of whole milk to optimize absorption[17]. Most doses were either administered in the clinic or observed during a home visit by a caregiver. For evening doses that were not observed, the timing of AL dosing was recorded in a log. During AL treatment, participants on NVP continued to receive the same NVP-based treatment prescribed by their physician for their HIV infection that they were receiving at time of malaria presentation. Adherence to the NVP-based regimen was monitored through patient logs. The study was approved by the Uganda National Council for Science and Technology, Makerere University School of Medicine Research and Ethics Committee for the site in Kampala, Uganda and the Ethics Committees for the sites in Lilongwe and Blantyre, Malawi. All participants or their guardians read and signed the informed consent before participation. The study was registered at Clinicaltrials.gov as NCT01728961and can be found at https://www.clinicaltrials.gov/ct2/show/NCT01728961?term=NCT01728961&rank=1. The protocol can be found at: http://impaactnetwork.org/studies/P1079.asp. This trial was registered at ClinicalTrials.gov by administrators of the Clinical Trials network (IMPAACT) and it was an oversight that it was not registered prior to initiating enrollment. Once the network was alerted to the need to register it, this was completed. Authors confirm that all ongoing and related trials for AL in this study are currently registered in Clinicaltrials.gov.

### HIV-uninfected control group

Due to low enrollment of HIV-infected children on no ART (see *Results*), PK results for this study were compared to an identically designed study of HIV-uninfected children, aged 5 to 12 years, also conducted in Kampala, Uganda[15]. Methods and results have been published previously. Briefly, eligibility criteria were identical to P1079, with children treated for their uncomplicated malaria with AL using the same PK design and weight-based dosing. PK sampling around the last dose of a 6 dose regimen was similar, with only the 1 hr and 264 hr (14 day) samples omitted in the historic controls.

### Safety monitoring

Management of adverse experiences was according to best clinical practice and judgment of the site investigator. All adverse events were classified according to the Division of AIDS Table for Grading the Severity of Adult and Pediatric Adverse Events, Version 1.0, dated December 2004, Clarification August 2009, available on the Regulatory Support Center (RSC) web site[16]. Abnormal clinical and laboratory findings were followed until resolution to < Grade 2.

### Pharmacokinetic sample collection and processing

Intensive serial PK sampling for artemether (ARM), its active metabolite dihydroartemisinin (DHA), and the long-acting partner drug LR, was conducted on study day 3 (prior to the last (6^th^) AL dose and post-last dose at 1, 2, 4, and 8 hr), day 4 (24 hr), day 8 (120 hr) and day 14 (264 hr). Venous blood samples were collected for determination of ARM, DHA and LR plasma concentrations. Blood samples were collected at 120 and 264 hr for analysis of LR only. Samples (0.5 mL) were drawn into EDTA-containing tubes which were immediately placed on ice and centrifuged at 800g for 10 minutes at 4°C. The resulting plasma was split into aliquots and kept at -70°C until analysis.

The dosing strategy in this study allowed sampling at 120 hr which technically occurred on day 8, but corresponds to day 7 results reported previously in the literature. Thus, this time point will be referred to as day 7 in the results.

For the control group, venous blood samples were collected from days 3 to 8, prior to the last AL dose, and post-dose at 2, 4, 8, 24, and 120 hr (also referred to as day 7). Three mL venous blood samples were drawn into sodium heparin tubes, centrifuged at 3,000xg for 15min at room temperature, and stored at -80°C until analysis.

### Plasma samples analysis

All samples were analyzed at Drug Research Unit at University of California, San Francisco. Liquid chromatography tandem mass spectrometry (LC-MS/MS) was used to measure ARM, DHA and LR, as previously described [18, 19]. Both methods were validated based on guidelines of Clinical Pharmacology Quality Assurance (CPQA) program sponsored by Division of AIDS at National Institute of Allergy and Infectious Diseases[20]. ARM and DHA were analyzed from 50 μL plasma samples. The lower limit of quantification (LLOQ) was 0.5 ng/mL and the calibration range was 0.5–200 ng/mL[18]. During sample analysis, the CV% for quality control (QC) samples was within 15% for intraday precision and <8% for interday precision for both ARM and DHA. LR concentrations were determined from 25 μL plasma samples. The calibration range was 50–20000 ng/mL, and the LLOQ was 50 ng/mL [19]. The CV% during sample analysis was within 15% for intraday precision and <11% for interday precision.

ARM, DHA and LR for the historical control HIV-uninfected children were analyzed in the Clinical Pharmacology Laboratory at the Mahidol-Oxford Tropical Medicine Research Unit in Thailand, using a nearly identical methodology on which our method was based [21]. Analytical specifications were described previously in detail with the LLOQs being 1.43, 1.43, and 25 ng/mL for ARM, DHA, and LR, respectively, and CVs being <5% (for ARM and DHA) and <6% (for LR)[15].

### Statistical methods

Safety was summarized in all enrolled P1079 participants and included any events occurring after the first AL dose, even if it was before study entry. Statistical comparisons for safety were not done because of the small number of participants not on ART. PK results were reported only in the NVP group and did not include one participant whose samples were not shipped to the testing lab. The primary outcomes for PK were AUC, peak concentration (C_max_) and time of peak concentration (T_max_) of ARM, DHA, and LR and the 7 day concentration for LR (C_day7_). Parameters were estimated with non-compartmental analysis using WinNonlin 6.2.1® (Certara L.P., Princeton, NJ, USA). Samples below the LLOQ were treated as missing except for the pre-dose drug concentration, which was set to 0 if below the LLOQ. AUC was calculated from 0 to 8hr (AUC_0-8hr_) for ARM and DHA, and from 0 to 120 hr (AUC_0-120hr_) for LR, to permit direct comparison between the NVP-treated children and the HIV-uninfected historical control children for whom the last data point was at 120hr [15]. AUC was calculated using the linear up-log down trapezoidal rule. Results for the control group were reanalyzed using identical PK analysis procedures.

Because of differences in distributions, AUC and C_max_ were summarized using geometric means (95% confidence intervals), and T_max_ and C_day7_ were summarized using medians (range). Distributions were compared between HIV-infected children on NVP-based ART and the HIV-uninfected controls using Wilcoxon rank sum tests. Differences were considered to be statistically significant if p<0.05. Analyses were conducted in SAS Version 9.4 (SAS Institute Inc, Cary, North Carolina, USA).

## Results

### Accrual and demographics

**Fig 1:**
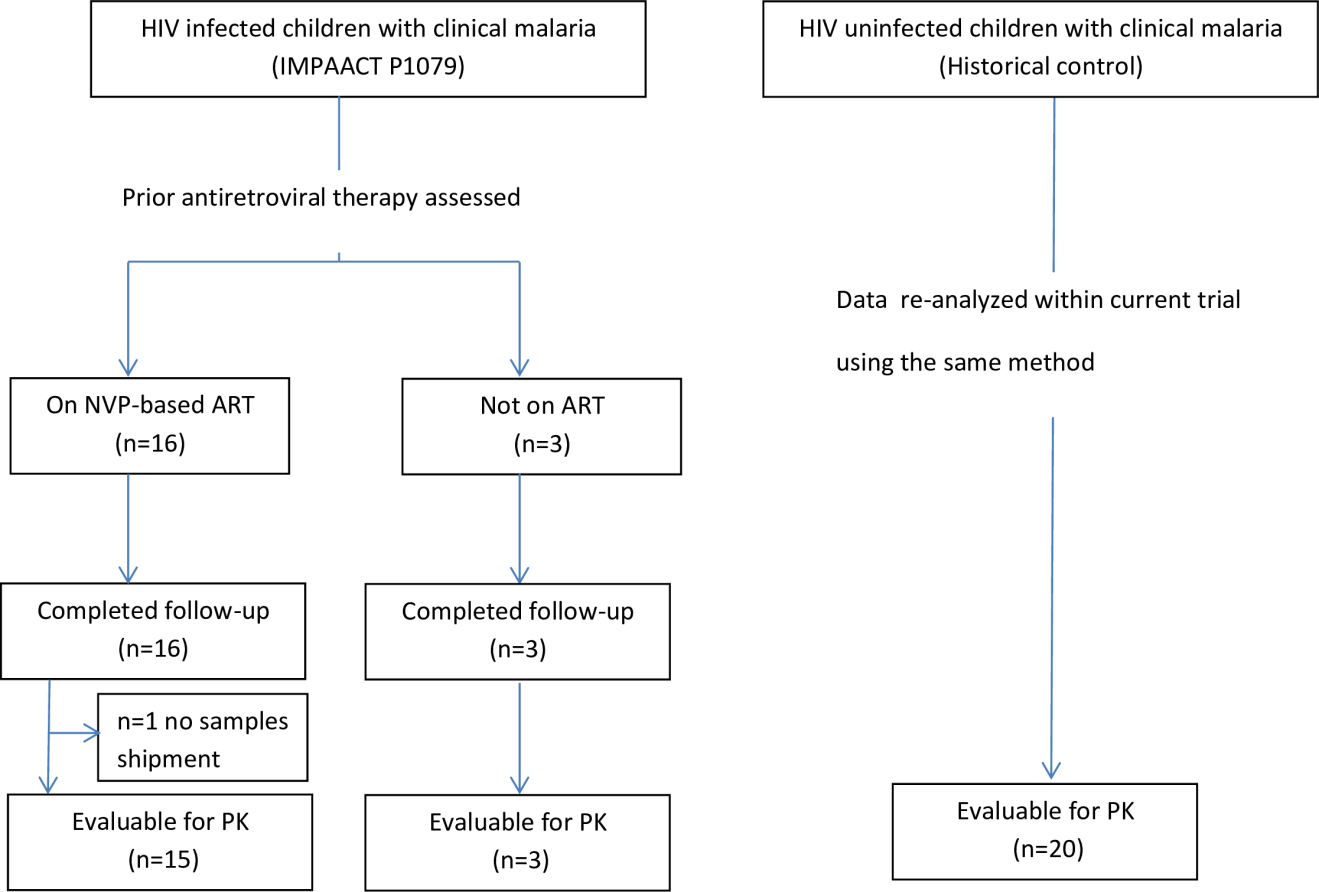
Enrollment and completion of intensive PK studies from a trial evaluating AL in children with or without NVP-based ART therapy.

Enrollment and study completion are shown in Fig 1. Due to low accrual and evolving WHO guidelines for treating HIV-infected children, enrollment was halted before reaching target accrual goals. Enrollment started on February 4, 2012 and the last follow up was completed in March 2014. For the NVP group, 9, 6, and 1 participants were enrolled in 2012, 2013, and 2014, respectively. For the no ART group, 3 participants were enrolled in 2012. Nineteen (40%) of the targeted 48 participants were enrolled with the majority of participants receiving NVP-based ART (n = 16) (Table 1). Of these, all were on NVP, lamivudine and zidovudine, except one child who was on NVP, lamivudine and stavudine. Six (38%) were female, median age was 7.1 years (range: 4.1–11.3 years), with a median weight of 17.9 kg (range: 14.0–29.3 kg). Only three HIV-infected children not on ART were enrolled. Median age was 8.3 years, median weight was 19.7 kg, and two were female.

**Table 1:** Demographics for P1079 participants and HIV-1 uninfected historical controls.

Characteristic		NVP	No ART	Historical controls
		(N = 16)	(N = 3)	(N = 20)
HIV-1 status		positive	positive	negative
Sex	Male	10 (63%)	1 (33%)	10 (50%)
	Female	6 (38%)	2 (67%)	10 (50%)
Age (yrs)	Median (range)	7.1 (4.1, 11.3)	8.3 (3.6, 10.0)	8.5 (5, 12)
Weight (kg)	Median (range)	17.9 (14.0, 29.3)	19.7 (16.1, 27.0)	25.5 (20.0, 41.0)

For the HIV-uninfected control group that served as historical controls, demographics have been reported previously[15]. Briefly, this group included 20 children: 50% were female, median age was 8.5 years (range: 5.0–12.0 years) and median weight was 25.5 kg (range: 20.0–41.0 kg).

### Safety

Of the 16 children on NVP, seven (44%: 95% confidence interval (CI): 20% - 70%) experienced at least one new grade ≥3 safety event: six low absolute neutrophil counts (ANC) (four grade 3 and two grade 4) and one recurrent malaria diagnosis with grade 3 fever. None (0%: 95% CI: 0% - 71%) of the HIV positive children not on ART had any new grade ≥3 safety events.

### Pharmacokinetic results

Samples for one participant at the Malawi site were never shipped, so the PK analysis included data on 15 children in the NVP-based ART group [5 females, median age 7.2 years (range: 4.1 to 11.3 years), median weight 19.4 kg (range: 14.0 to 29.3 kg)].

#### Impact of NVP on ARM and DHA disposition

**Fig 2:**
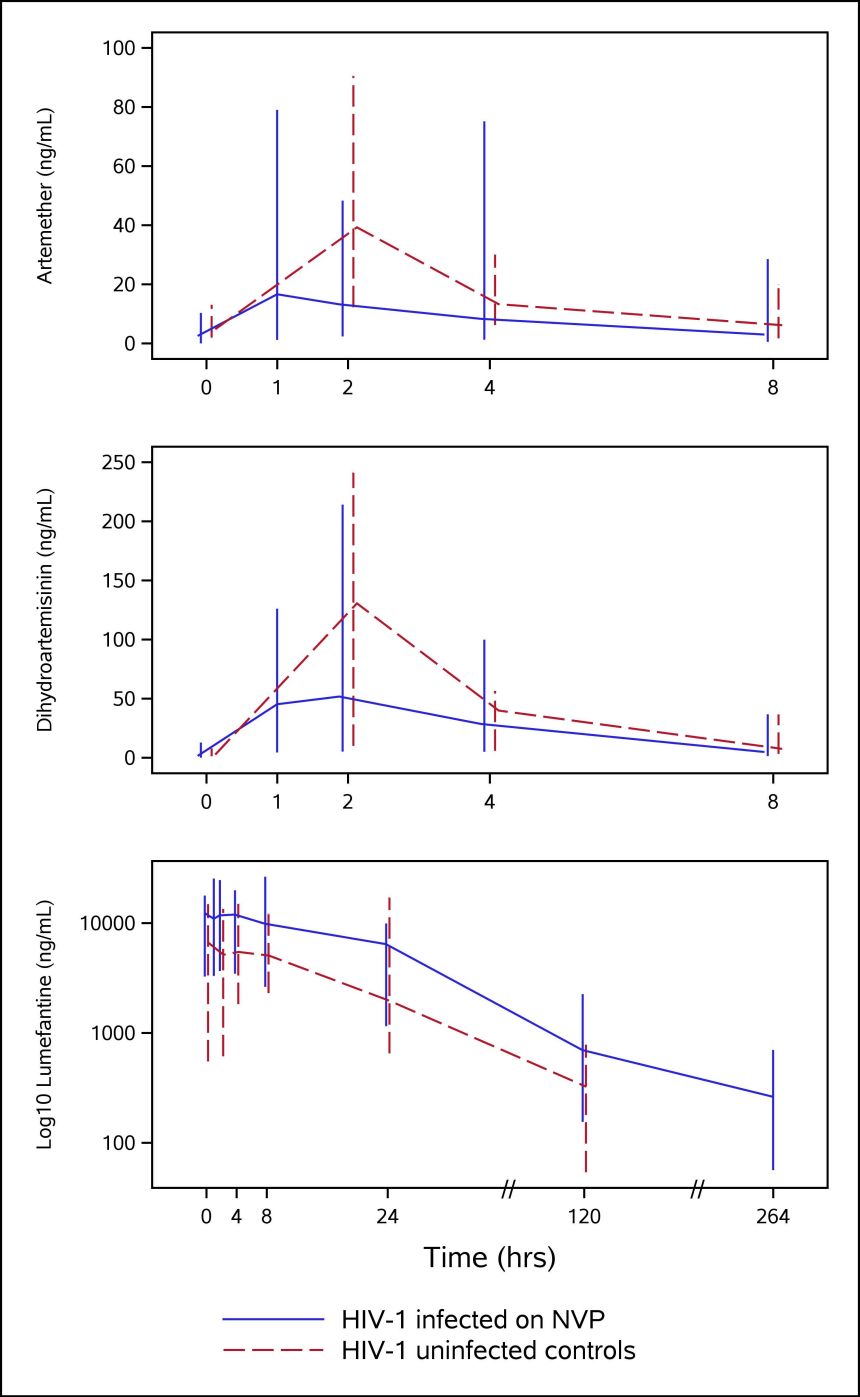
Median (min, max) plasma concentration versus time profile for artemether, dihydroartemisinin and lumefantrine (log_10_ scale) after AL administration alone in HIV-uninfected controls (dashed line) and with NVP in HIV-infected P1079 participants (solid line).

PK results for ARM, DHA and LR are summarized in Table 2. Compared to the HIV-uninfected controls, co-administration of NVP in HIV-infected children was associated with a trend toward decreased C_max_ and AUC_0-8hr_ for both ARM and DHA. The differences in ARM were larger: C_max_ decreased by 50% for ARM (p = 0.07) and by 36% for DHA (p = 0.01); AUC_0-8hr_ decreased by 46% for ARM (p = 0.08) and 22% for DHA (p = 0.06). Decreased T_max_ (35%, p = 0.028) was only observed for ARM. Not all differences were statistically significant (p<0.05), but the trend of decreased exposure with co-administration of NVP was evident. Median (range) plasma concentration-time profiles of ARM and DHA are shown in the first two panels of Fig 2.

**Table 2:** PK parameters for ARM, DHA, and LR after administration of AL in HIV-infected children on NVP compared to HIV-uninfected children (historical controls).

	AL+NVP	AL (controls)	Difference
	n = 15	n = 20*	% (p-value)
**ARM**			
C_max_ (ng/mL)	17.2 (9.9 to 29.9)	34.4 (27.1 to 43.8)	-50.0 (0.07)
T_max_ (hr)	1.3 (1.0 to 4.0)	2.0 (1.8 to 8.0)	-35 (0.028)
AUC_0-8hr_ (hr•ng/mL)	65.9 (38.1 to 114)	123 (99.2 to 153)	-46.4 (0.08)
**DHA**			
C_max_ (ng/mL)	76.5 (57.6 to 101.4)	119 (96.7 to 147)	-35.7 (0.01)
T_max_ (hr)	2.0 (1.0 to 4.0)	2.0 (1.8 to 8.0)	0.0 (0.12)
AUC_0-8hr_ (hr•ng/mL)	261 (206 to 330)	336 (279 to 404)	-22.3 (0.06)
**LR**			
C_max_ (μg/mL)	13.2 (10.3 to 17.0)	6.76 (5.36 to 8.52)	95.3 (<0.001)
T_max_ (hr)	2.0 (0.0 to 8.0)	4.0 (0.0 to 24.2)	-50.0 (0.96)
AUC_0-120hr_ (hr•μg/mL)	435 (333 to 569)	195 (150 to 253)	123 (<0.001)
C_day7_ (ng/mL)	697 (155 to 2250)	323 (53.9 to 779)	116 (<0.001)

AUC and C_max_ summarized using geometric means (95% confidence intervals)

T_max_ and C_day7_ summarized using medians (range).

Statistical comparisons between groups from Wilcoxon rank sum tests

#### Impact of NVP on LR disposition

In contrast to results for ARM and DHA, NVP was associated with a significant increase on LR exposure as measured by C_max_ (95%, p<0.001) and AUC_0-120hr_ (123%, p<0.001). Day 7 LR concentrations were also higher by 116% (p<0.001) (median C_day7_ in the NVP group was 697 ng/mL versus 323 ng/mL in the controls) (Table 2). The median (range) plasma concentration-time profile of LR is shown in the last panel of Fig 2.

## Discussion

This study in children on stable NVP-based ART showed that NVP had a significant impact on the PK disposition of LR and marginal impact on ARM and DHA, when AL was used for treatment of uncomplicated malaria. Compared to a group of HIV-uninfected children (previously reported) with a similar age range (5.0–12.0 years for controls versus 4.1–11.3 years for children on NVP-based ART), HIV-infected children on NVP had reductions in the exposure of ARM and DHA but increased exposure to LR. The findings in this study are consistent with results reported by Kredo et al.[14] and Maganda et al.[10] but different from two other studies in Nigerian and Ugandan adults [9, 13]. Differences in ARM, DHA, and LR exposure in these studies are summarized in Table 3. Geometric mean changes are quite different from median changes for LR in our study, but since four other published studies reported medians, we also reported median differences. Specifically, there were statistically significant lower median peak concentrations for DHA (38%, p = 0.01), and significantly higher median peak concentrations (106%, p<0.001), AUC_0-120h_ (206%, p<0.001), and C_day7_ (116%, p<0.001) for LR. There were also trends toward decreases in AUC for ARM (48%, p = 0.08) and DHA (18%, p = 0.06). The study by Kredo et al. used a similar design but in HIV-infected adults with intensive PK sampling starting post-1^st^ AL dose. Between 60-68hr (after the 6^th^ AL dose), corresponding to 0-8hr in our study, the NVP-treated group had a reduction in ARM C_max_ (56%, p<0.015) and AUC (55%, p = 0.12). DHA exposure was reduced to a lesser extent (C_max_ 14%, p = 0.08, AUC 25%, p = 0.01). Consistent with our current study, LR exposure was also increased with NVP by 85% for day 7 concentration (median day 7 LR concentration of 622 ng/mL with NVP vs 336 ng/mL in those not on NVP, p<0.001) and 56% for AUC_0-∞_ (693 hr.μg/mL with NVP vs 445 hr.μg/mL in those not on NVP, p = 0.001). Likewise, Maganda et al. studied adults and also reported an increased exposure of day 7 LR concentrations (1125 versus 970 ng/mL, p = 0.063)[10]. In contrast, Byakika-Kibwika et al reported reduced exposure to all drug components for AL in adults. NVP led to significantly reduced exposure of ARM and DHA including peak concentration and AUC, where median AUC was reduced by 72% and 37%, respectively, but where the reduction in LR exposure was not significant [9]. A recently reported study in HIV-infected Nigerian adults also compared to a historical control group, NVP reduced exposure of ARM and LR significantly, but not DHA exposure [13]. In this study LR samples were only collected up to 96hr, and drugs were measured with less sensitive methods[22, 23]. Lastly, our group has recently evaluated this interaction in younger children (6 months to 6 years of age) in Tororo Uganda, a region highly endemic for malaria. NVP reduced AUC_0-8hr_ for both ARM and DHA by 68% (p<0.0001), and 28% (p = 0.02), respectively, but only led to a small trend toward increased LR exposure of 18% (p = 0.8) based on median AUC, the geometric mean AUC changes are -70%, -35%, and +3% for ARM, DHA, and LR, respectively[24].

**Table 3:** Comparison of published AL exposure in the context of NVP-based ART therapy.

	This study			Kredo et al. 2011		Maganda et al. 2014		Kibwika et al, 2012		Parikh et al. 2015	
	median change %	geomean change %	P value	median change%	p value	median change%	p value	median change%	p value	geomean change%	p value
ARM_Cmax	-54	-50	0.07	-56	0.015			-61	<0.01	-68	<0.01
ARM_AUC	-48	-46	0.08	-55	0.12			-72	<0.01	-68	<0.01
DHA_Cmax	-38	-36	0.01	-14	0.08			-45	<0.01	-23	0.37
DHA_AUC	-18	-22	0.06	-25	0.01			-37	<0.01	-11	0.69
LR_Cmax	106	95	<0.001	24	0.06			-24	0.6	-47	0.07
LR_AUC	206	123	<0.001	56	0.001			-21	0.4	-49	0.048
LR_C_day7_	116	172	<0.001	85	<0.001	16	0.063	-9.6	0.2		

Reasons for discrepancies on LR exposure between studies are unclear, but could be attributed to differences in study design. This study along with the other two studies by Kredo et al and Maganda et al used parallel designs, while Byakika-Kibwika et al used a cross-over design. Although there was a 50-day washout period between the 2 periods of AL administration in the cross-over study, the effect of artemisinin induced CYP enzymes change on LR metabolism is unclear. Parikh et al. used less PK sampling points (up to 96hr only) and a historical control. We also relied on historical control data in HIV-1 uninfected children, which used a nearly identical design in that intensive PK was collected around the last dose of a 6 dose regimen. However, when comparing results for the NVP-treated children to the three HIV-1 infected children enrolled into P1079 not on ART, the same patterns were evident, with a trend towards reduced exposure for ARM and DHA and increased exposure for LR (70 vs 66 hr.ng/mL for ARM AUC, 265 vs 261 hr.ng/mL for DHA AUC, 383 vs 435hr.μg/mL for LR AUC, 528 vs 697 for LR C_day7_ for the no ART vs NVP groups respectively, supporting information S1 Table). Differences in median AUCs were larger: 106 vs 64.5 for ARM, 381 vs 279 for DHA, and 368 vs 549 for LR).

NVP is extensively metabolized by CYP3A4 and CYP2B6[16], and in addition to inductive effects, it can serve as a competitive inhibitor of these enzymes in the presence of other substrate drugs[25]. We speculate impact from CYP enzyme induction will be dominant for fast metabolizing drugs like artemisinins, as artemisinins are better substrates than NVP. But for the slow metabolizing drug LR, NVP will competitively bind to CYP3A4 to inhibit metabolism of LR, which explains the decreased exposure of ARM and DHA, and increased exposure of LR in the context of NVP. Moreover clinically, NVP has been shown to inhibit CYP3A4 metabolism, e.g. a 1.5-fold higher C_max_ of maraviroc, also a CYP3A4 substrate[26] was observed in participants on NVP-based ART compared to historical controls receiving maraviroc monotherapy[27].

AL has been proved to be safe with minimal side effects[28]. In this study we evaluated safety of AL in the context of NVP. The proportion of participants experiencing grade ≥3 safety events in the HIV-infected children on NVP was 44% (n = 16) compared to 0% (n = 3) for those not on ART, but with the small sample sizes, there was inadequate power to detect other than very large differences.

This study has limitations, including a small sample size, use of a historical control group of HIV-uninfected children who were somewhat older and heavier than the NVP-based ART group in this report, and children with only uncomplicated malaria. HIV infection itself might impact AL PK, and there could have been other differences between the cohorts. Because of the small sample size, we could not conduct analyses adjusted for other factors. Assessing adherence to study drugs is also a challenge. Although we observed dosing before PK sampling, we relied on patients’ log for some doses of AL and ART. More advanced techniques are available to improve adherence, such as high-tech pill bottles, which can count number of times bottle opened. Although the study protocol required AL was taken with whole milk, milk may not be available in some resource-limited communities, alternative fatty food such as oil-fortified porridge might be used without compromising drug absorption[29]. In addition, in this study we did not measure the NVP concentrations, which would be helpful to verify these children had therapeutic NVP levels.

Most recent WHO guidelines recommend the use of LPV/r -based ART in children under 3 years of age and efavirenz-based ART in those above 3 years of age, with NVP-based ART as an alternate regimen[6]. However, due to delayed uptake and availability, NVP use in Africa remains high[30, 31]. Thus it remains important to ensure the proper use of antimalarials in the context of NVP treatment. Increase in LR exposure in this study may not be a concern. Increased exposure of LR with LPV/r ART has also been reported with much more extensive increases in exposure [8]. Due to the excellent safety profile of AL, dosage adjustment even with these increases, has not been recommended [32]. However, the reductions in the artemisinins with NVP as reported here may of concern and warrant further study to determine the correct dose in young children. Alteration of AL exposure may alter clinical outcomes[33]. A recent meta-analysis found a higher ARM dose was associated with a lower gametocyte presence within 14 days of treatment and authors suggested a higher AL dose should be accessed in young children[34]. Suboptimum dosing of AL in children will contribute selection of resistance to this drug. Considering reports of artemisinin resistance in Southeast Asia[35–37], it is important to optimize AL dose in children.

In summary, in this study we showed that NVP use in children 4–12 years of age was associated with trends of reduced exposure of both ARM and DHA and significantly increased exposure of LR when compared to HIV-1 uninfected controls. Considering the excellent safety profile of AL, the increase in LR is not of concern. However, the reduction in artemisinin exposure may warrant further study, and suggests that dosage adjustment of AL with NVP based ART in children is likely warranted.

## Supporting information

S1 Text TREND checklist. (PDF)

S2 Text P1079 protocol. (PDF)

S1 Table PK parameters compared to the 3 control participants from P1079. (XLSX)

S2 Table Characteristics of P1079 participants at study entry. (RTF)
