# Supplementary material for: Concomitant nevirapine impacts pharmacokinetic exposure to the antimalarial artemether-lumefantrine in African children
Source: PLoS One. 2017 Oct 24;12(10):e0186589. doi: 10.1371/journal.pone.0186589 (PMC5655345; doi:10.1371/journal.pone.0186589)
Supplement: S2 Text — (PDF) [file pone.0186589.s002.pdf]

IMPAACT P1079
Pharmacology of Artemisinin-based Antimalarial Therapy within the Context of Antiretroviral Therapy

DAIDS ID #: 10765

This file contains the current IMPAACT P1079 protocol, which is comprised of the following documents, presented in reverse chronological order:

- Letter of Amendment #2, dated 6 December 2011
- Letter of Amendment #1, dated 6 January 2011
- Clarification Memo #1, dated June 2010
- Protocol Version 1.0, dated 21 May 2010

TO:	IMPAACT Principal Investigators & Study Coordinators at Sites Participating in P1079
FROM:	P1079 Protocol Team
DATE:	December 06, 2011
RE:	Letter of Amendment #2 for Protocol P1079, Version 1.0, dated May 21, 2010, entitled "Pharmacology of Artemisinin-Based Antimalarial Therapy within the Context of Antiretroviral Therapy"

THE FOLLOWING INFORMATION IMPACTS THE P1079 STUDY AND MUST BE FORWARDED TO YOUR INSTITUTIONAL REVIEW BOARD (IRB)/ETHICS COMMITTEE (EC) AS SOON AS POSSIBLE FOR THEIR REVIEW. THIS LETTER OF AMENDMENT MUST BE APPROVED BY YOUR IRB/EC BEFORE IMPLEMENTATION.

THE FOLLOWING INFORMATION MAY IMPACT THE SAMPLE INFORMED CONSENT. YOUR IRB/EC WILL BE RESPONSIBLE FOR DETERMINING THE PROCESS OF INFORMING SUBJECTS OF THE CONTENTS OF THIS LETTER OF AMENDMENT.

UPON RECEIVING FINAL IRB/EC AND ANY OTHER APPLICABLE REGULATORY ENTITY (RE) APPROVAL(S) FOR THIS LOA, SITES SHOULD IMPLEMENT THE LOA IMMEDIATELY. SITES ARE STILL REQUIRED TO SUBMIT A LOA REGISTRATION PACKET TO THE DAIDS PROTOCOL REGISTRATION OFFICE (DAIDS PRO) AT THE REGULATORY SUPPORT CENTER (RSC). SITES WILL RECEIVE A REGISTRATION NOTIFICATION FOR THE LOA ONCE THE DAIDS PRO VERIFIES THAT ALL THE REQUIRED LOA REGISTRATION DOCUMENTS HAVE BEEN RECEIVED AND ARE COMPLETE. A LOA REGISTRATION NOTIFICATION FROM THE DAIDS PRO IS NOT REQUIRED PRIOR TO IMPLEMENTING THE LOA. A COPY OF THE DAIDS PRO LOA REGISTRATION NOTIFICATION ALONG WITH THIS LETTER AND ANY IRB/EC CORRESPONDENCE SHOULD BE RETAINED IN THE SITE'S REGULATORY FILES.

This letter of amendment can be obtained from the P1079 protocol-specific web page (https://impaactgroup.org/); Enter the Member/MIS area using your individual username and password. Search for the study number. From the P1079 web page you will have the option to click the PSWP tab to retrieve the document.

This Letter of Amendment (LOA) serves to inform sites of the changes to:

Section 6.211, Appendix I (Schedule of Evaluations), and the sample informed consent (SIC). Blood for the thick and thin blood smears for diagnosis of malaria parasitemia can be collected in an EDTA-containing vacutainer tube when venous blood is being collected for other laboratory evaluations (principally hematology or pharmacokinetic samples). All other thick and thin blood smear collections should be performed via finger stick.

- The first sentence of Section 6.211 is modified as follows:

  Subjects will be referred to the outpatient laboratory for screening thick blood smear, obtained by fingerprick **or from blood collected in an EDTA-containing vacutainer tube when venous blood is being collected for other laboratory evaluations (principally hematology or pharmacokinetic samples)**, using standard Giemsa staining.

- Appendix I (Schedule of Evaluations) is modified to remove the reference to finger stick in the table for thick and thin blood smears and to include the following footnote:

  Blood for the thick and thin blood smears for diagnosis of malaria parasitemia can be collected in an EDTA-containing vacutainer tube but ONLY when venous blood is being collected for other laboratory evaluations (principally hematology or pharmacokinetic samples). All other thick and thin blood smear collections should be performed via finger stick.

  Please see modified version of Appendix I attached to this document.

- Sample informed consent (SIC), WHAT DOES MY CHILD HAVE TO DO IF HE/SHE IS IN THIS STUDY? section: The visit table in this section is modified as follows:

	Study Day 0 (Screening & Entry)	Study Day 1	Study Day 2	Study Day 3	Study Day 4	Study Day 8	Study Day 14	Study Day 28	Study Day 42 End of study	Unscheduled Sick Visit	Early Discontinuation
Informed Consent	X										
History	X	X	X	X		X	X	X	X	X	X
Physical exam	X	X	X	X		X	X	X	X	X	X
Coartem® Dispersible given at Clinic	X	X	X	X							
Coartem® Dispersible given at home*	X		X								
Test for malarial infection. **Blood may be taken by finger prick.**	X	X	X	X	X	X	X	X	X	X	X
Blood Collected	X			X	X	X	X	X	X		X
Pregnancy test**	X										

- Sample informed consent (SIC), WHAT DOES MY CHILD HAVE TO DO IF HE/SHE IS IN THIS STUDY? section, sub-section Before starting this study ("Screening Visit"): The second item under the 4th bullet point has been deleted and a 5th bullet point has been added. This section is modified as follows:

  After you have read and signed this consent form, your child will have the following done at the screening visit to see if your child can participate in the study:
  - You will be asked questions about your child's medications (anti-HIV and anti-malarial) and medical history. You may be asked for permission to review your child's medical record.
  - Your child will have a complete physical exam including height, weight, and vital signs (temperature, blood pressure, pulse, and respiratory rate).
  - If your child is female and has had her first menstrual period, your child will provide a urine or blood sample for a pregnancy test. The test must show that your child is not pregnant in order for your child to participate in this study.
  - Your child will have about ½ teaspoon (2.5 mL) of blood drawn:
    - for routine blood tests,
    - to measure your child's HIV viral load (how much HIV is in your child's blood), and
    - to test for HIV resistance to HIV drugs (changes in your child's HIV)
  - **Your child will have a small amount of blood taken to check for the amount of malarial parasites. This blood may be taken by a finger prick.**

  The results of these tests will determine if your child is eligible to enter the study.

- Sample informed consent (SIC), WHAT DOES MY CHILD HAVE TO DO IF HE/SHE IS IN THIS STUDY? section, sub-section Entry Visit (Study Day 0): The 4th bullet point in this section is modified as follows:

  - **Your child will have a small amount of blood taken to check for the amount of malarial parasites. This blood may be taken by a finger prick.**

- Sample informed consent (SIC), WHAT DOES MY CHILD HAVE TO DO IF HE/SHE IS IN THIS STUDY? section, sub-section Study Day 3: The 3rd bullet point in this section is modified as follows:

  - **Your child will have a small amount of blood taken to check for the amount of malarial parasites. This blood may be taken by a finger prick.**

- Sample informed consent (SIC), WHAT DOES MY CHILD HAVE TO DO IF HE/SHE IS IN THIS STUDY? section, sub-section Study Day 4: The 2nd bullet point in this section is modified as follows:

  - **Your child will have a small amount of blood taken to check for the amount of malarial parasites. This blood may be taken by a finger prick.**

- Sample informed consent (SIC), WHAT DOES MY CHILD HAVE TO DO IF HE/SHE IS IN THIS STUDY? section, sub-section Study Day 8: The 3rd bullet point in this section is modified as follows:

  - **Your child will have a small amount of blood taken to check for the amount of malarial parasites. This blood may be taken by a finger prick.**

- Sample informed consent (SIC), WHAT DOES MY CHILD HAVE TO DO IF HE/SHE IS IN THIS STUDY? section, sub-section Study Days 14, 28, and 42: The 3rd bullet point in this section is modified as follows:

  - **Your child will have a small amount of blood taken to check for the amount of malarial parasites. This blood may be taken by a finger prick.**

- Sample informed consent (SIC), WHAT DOES MY CHILD HAVE TO DO IF HE/SHE IS IN THIS STUDY? section, sub-section Early discontinuation from the study: The 3rd bullet point in this section is modified as follows:

  - **Your child will have a small amount of blood taken to check for the amount of malarial parasites. This blood may be taken by a finger prick.**

These updates will be made in the next version of the protocol when it is amended. Please contact the protocol team at actg.teamp1079@fstrf.org if you have any questions. Thank you for your interest in IMPAACT P1079.

Thank you for your participation in IMPAACT P1079.

APPENDIX I
SCHEDULE OF EVALUATIONS

	Scenario #1: Separate Screening and Entry Visits		OR	Scenario #2: Combined Screening and Entry Visits										
	Screening [1, 2]	Study Day 0 Entry[2,3]		Screening/ Study Day 0 Entry [1, 2,3]	Study Day 1[2]	Study Day 2[2]	Study Day 3	Study Day 4	Study Day 8 (+24 hour window)[4]	Study Day 14 (+24 hour window)[4]	Study Day 28	Study Day 42 End of study	Unscheduled Sick Visit	Early Discontinuation[21]
CLINICAL EVALUATIONS														
Informed Consent	X			X										
History[5]	X	X		X	X	X	X		X	X	X	X	X	X
Physical exam[6]	X	X		X	X	X	X		X	X	X	X	X	X
Adherence Assessment[7]	X	X		X	X	X	X							
Artemether-lumefantrine (AL) dosing[8]		Dose 1- clinic Dose 2- home		Dose 1- clinic Dose 2- home	Dose 3- clinic	Dose 4- clinic Dose 5-home	Dose 6- clinic[9]							
LABORATORY EVALUATIONS														
Hematology[10]	1 mL			1 mL						1 mL	1 mL	1 mL		1 mL
Chemistries[11]	1 mL			1 mL						1 mL	1 mL	1 mL		1 mL
Thick Blood Smear[12]	0.05	0.05		0.05	0.05	0.05	0.05		0.05	0.05	0.05	0.05	0.05	0.05
Thin Blood Smear[12]	0.05			0.05										
Dried Blood Spots (DBS) for Malaria Infection[13]		0.1 mL		0.1 mL	0.1 mL	0.1 mL	0.1 mL	0.1 mL	0.1 mL	0.1 mL	0.1 mL	0.1 mL	0.1 mL	0.1 mL
Urine [or Blood] Pregnancy test [14]	2 mL [1-2 mL] [18]	2 mL[17] [1-2 mL] [18]		2 mL [1-2 mL] [18]										
Virology														
DBS for Viral Load[15]	0.25 mL	0.25 mL[17]		0.25 mL					0.25 mL	0.25 mL		0.25 mL		0.25 mL
Drug Resistance Testing (DBS)[16]	0.25 mL	0.25 mL[17]		0.25 mL								0.25 mL		0.25 mL
Pharmacology														
Artemether-lumefantrine (AL) PK[19]							15 mL	3 mL	3 mL	3 mL				
Nevirapine PK[20]		1 mL		1 mL			1 mL							
TOTAL BLOOD VOLUMES	2.6-4.6 mL	1.15-3.65 mL		3.7-5.7 mL	0.15 mL	0.15 mL	16.15 mL	3.1mL	3.4 mL	5.4 mL	2.15 mL	2.65 mL	0.15 mL	2.65 mL

APPENDIX I (Cont.)

1. Screening evaluations must be performed within 30 days of Entry.
2. Note that screening and entry can occur on the same day. In addition, for subjects who have started AL prior to study consent and have received up to 3 doses of AL, study Days 0, 1 and 2 evaluations may all be collapsed into a single visit, consistent with all criteria listed on study Day 0 (entry visit). Subjects who enter the study after having initiated AL may have a study Day 0 visit, with the next visit matched to the subject's day of AL treatment.
3. Subjects with Grade 3 or 4 hemoglobin at entry are allowed to participate in all study procedures (e.g. evaluation of the 28 and 42-day safety and toxicity) EXCEPT for the AL PK sampling or single samples for NVP measurements (to mimimize the volume of blood drawn in these children).
4. Team should be notified within 24 hours if timelines (i.e. +24 window) can not be met.
5. A complete history is required at Screening/Entry Visit; a targeted history [i.e. review of new illnesses and/or symptoms] is sufficient at subsequent visits.
6. Complete physical exam (including height, weight, vital signs [temperature, pulse, respiration, and blood pressure]) at Screening/Entry Visit and targeted physical exam (including vital signs [temperature, pulse, respiration, and blood pressure]) is sufficient at all other visits.
7. Adherence assessment will be conducted both retrospectively for the doses AL already taken, as well as, prospectively. All doses require documentation of date and time.
8. Dosing of AL must be performed according to schedule included in Section 5.1.
9. Draw Pre-Dose PK sample, then administer Dose 6 (the last dose) of AL.
10. Hematology should include: CBC.
11. Chemistries should include: AST, ALT, and serum creatinine.
12. Blood for the thick and thin blood smears for diagnosis of malaria parasitemia can be collected in an EDTA-containing vacutainer tube but ONLY when venous blood is being collected for other laboratory evaluations (principally hematology or pharmacokinetic samples). All other thick and thin blood smear collections should be performed via finger stick.
13. See section 6.214. Prepare 1 card with 2 spots at 0.05 mL each spot. Batched and shipped according to the instructions provided in the Laboratory Processing Chart (LPC). Note: DBS blood can be collected in a vacutainer tube ONLY when blood is collected for other laboratory evaluations (i.e. hematology, chemistry, or pharmacokinetic). All other DBS collections should be performed via finger stick. See Appendix III for additional collection, processing, and storage instructions.
14. Must be performed within 72 hours of enrollment on female subjects who have had their first menstrual period.
15. Prepare 1 card with 5 spots at 0.05 mL each spot. Batched and must be performed at DAIDS VQA-certified laboratory. Note: DBS blood can be collected in a vacutainer tube ONLY when blood is collected for other laboratory evaluations (i.e. hematology, chemistry, or pharmacokinetic). All other DBS collections should be performed via finger stick. See Appendix III for additional collection, processing, and storage instructions.
16. Prepare 1 card with 5 spots at 0.05 mL each spot. Collect on all study participants. To be performed on subjects who show changes in viral load pattern between study Day 0 and study Day 42 using dried blood spot collected at screening and study Day 42. Note: DBS blood can be collected in a vacutainer tube ONLY when blood is collected for other laboratory evaluations (i.e. hematology, chemistry, or pharmacokinetic). All other DBS collections should be performed via finger stick. See Appendix III for additional collection, processing, and storage instructions.
17. This evaluation should only be performed if it was not performed at Screening.
18. Blood pregnancy test can be substituted for urine pregnancy test.
19. AL pharmacokinetics (3 mL per sample) will be collected on study Day 3: Pre-dose, 1, 2, 4, and 8 hours; study Day 4: 24 hours; study Day 8: 120 hours post-administration of the last AL dose; and study Day 14: 264 hours post-administration of the last AL dose. Participants will be asked to remain within the clinic or research facility until completion of the 8 hour blood sample collection at which time they will be discharged for home. They will be asked to return to the clinic or research facility for the 24 hour (study Day 4) and 120 hour (study Day 8) blood sample collections. For subjects who experience treatment failure before study Day 3, PK sampling will be discontinued. Specimen aliquots are prepared according to instructions provided in the LPC.
20. NVP pharmacokinetic samples will be collected on study Day 0 and study Day 3. The baseline (study Day 0) pharmacokinetic samples of NVP will be collected at random times prior to initiating AL treatment and following the previous NVP dose (approximately 12 hours following the evening dose of NVP). The second sample, study Day 3, will be collected just prior to the morning NVP dose and at the time of the last dose of AL. Specimen aliquots are prepared according to instructions provided in the LPC.
21. Subjects who prematurely discontinue study treatment will continue to be followed at scheduled study visits, but only the evaluations listed in this column are required.

For insufficient blood draws at SCREENING visit, priorities are as follows:

a. Hematology
b. Chemistries
c. Nevirapine PK

For insufficient blood draws at DAY 3 visit, priorities are as follows:

a. Artemether-lumefantrine (AL) PK
b. Nevirapine PK

TO:	IMPAACT Principal Investigators & Study Coordinators at Sites Participating in P1079
FROM:	P1079 Protocol Team
DATE:	January 6, 2011
RE:	Letter of Amendment #1 for Protocol P1079, Version 1.0, dated May 21, 2010, entitled "Pharmacology of Artemisinin-Based Antimalarial Therapy within the Context of Antiretroviral Therapy"

THE FOLLOWING INFORMATION IMPACTS THE P1079 STUDY AND MUST BE FORWARDED TO YOUR INSTITUTIONAL REVIEW BOARD (IRB)/ETHICS COMMITTEE (EC) AS SOON AS POSSIBLE FOR THEIR REVIEW. THIS LETTER OF AMENDMENT MUST BE APPROVED BY YOUR IRB/EC BEFORE IMPLEMENTATION.

THE FOLLOWING INFORMATION MAY IMPACT THE SAMPLE INFORMED CONSENT. YOUR IRB/EC WILL BE RESPONSIBLE FOR DETERMINING THE PROCESS OF INFORMING SUBJECTS OF THE CONTENTS OF THIS LETTER OF AMENDMENT.

UPON RECEIVING FINAL IRB/EC AND ANY OTHER APPLICABLE REGULATORY ENTITY (RE) APPROVAL(S) FOR THIS LOA, SITES SHOULD IMPLEMENT THE LOA IMMEDIATELY. SITES ARE STILL REQUIRED TO SUBMIT A LOA REGISTRATION PACKET TO THE DAIDS PROTOCOL REGISTRATION OFFICE (DAIDS PRO) AT THE REGULATORY SUPPORT CENTER (RSC). SITES WILL RECEIVE A REGISTRATION NOTIFICATION FOR THE LOA ONCE THE DAIDS PRO VERIFIES THAT ALL THE REQUIRED LOA REGISTRATION DOCUMENTS HAVE BEEN RECEIVED AND ARE COMPLETE. A LOA REGISTRATION NOTIFICATION FROM THE DAIDS PRO IS NOT REQUIRED PRIOR TO IMPLEMENTING THE LOA. A COPY OF THE DAIDS PRO LOA REGISTRATION NOTIFICATION ALONG WITH THIS LETTER AND ANY IRB/EC CORRESPONDENCE SHOULD BE RETAINED IN THE SITE'S REGULATORY FILES.

This letter of amendment can be obtained from the P1079 protocol-specific web page (https://impaactgroup.org/); Enter the Member/MIS area using your individual username and password. Search for the study number. From the P1079 web page you will have the option to click the PSWP tab to retrieve the document.

This Letter of Amendment (LOA) serves to inform sites of the changes to:

- Section 3.2 (AL Dosing and Pharmacokinetics): The addition of AL PK sample for study Day 14. The paragraph in this section is modified as follows:

  Subjects who test positive for malaria will be given a total of 6 doses of AL, distributed over study Day 0 to study Day 3. Specifically, children will receive 2 weight-based doses of AL, approximately 8-12 hours apart on the day of study entry (study Day 0) and on study Day 2 of the study. On study Days 1 and 3, subjects will be given AL once daily. See Section 5.1 for AL dosing schedule. During AL treatment, subjects will also continue to receive the same NVP- based treatment prescribed by their physician (if any) for their HIV infection that they were receiving at time of malaria presentation. AL PK samples will be collected on study Day 3: Pre-dose, 1, 2, 4, and 8 hours; study Day 4: 24 hours; study Day 8: 120 hours post-administration of the last AL dose**; and study Day 14: 264 hours post-administration of the last AL dose**.

- Section 9.211 (AL Sampling): The addition of AL PK sample for study Day 14. The paragraph in this section is modified as follows:

  Pharmacokinetics of AL will be collected around the last treatment dose for all subjects enrolled in the study to compare the PK of AL in subjects who are receiving a NVP-based regimen for their HIV to subjects who are not receiving any ARVs for their HIV (Primary Objective 2.11). Plasma samples will be collected for AL PK on study Day 3: Pre-dose, 1, 2, 4, and 8 hours; study Day 4: 24 hours; study Day 8: 120 hours post-administration of the last AL dose**; and study Day 14: 264 hours post-administration of the last AL dose**.

- Appendix I (Schedule of Evaluations) regarding the *Dried Blood Spots (DBS) for Malaria Infection* and the *Artemether-lumefantrine (AL) PK* collection time points.
  - Dried Blood Spots (DBS) for Malaria Infection must also be collected at the following visits: Study Days 0, 1, 2, 3, 42, *Unscheduled Sick Visit*, and *Early Discontinuation*, in addition to the current visits (Study Days 4, 8, 14, and 28).
  - The first sentence in footnote 12 has been deleted. DBS for malaria infection must be collected for all subjects.
  - Artemether-lumefantrine (AL) PK must also be collected at the Study Day 14 visit.

  Please see modified version of Appendix I attached to this document.

- Sample informed consent (SIC), WHAT DOES MY CHILD HAVE TO DO IF HE/SHE IS IN THIS STUDY? section: The DBS for malaria infection must be collected for all subjects on Study Day 4. This visit table in this section is modified as follows:

	Study Day 0 (Screening & Entry)	Study Day 1	Study Day 2	Study Day 3	Study Day 4	Study Day 8	Study Day 14	Study Day 28	Study Day 42 End of study	Unscheduled Sick Visit	Early Discontinuation
Informed Consent	X										
History	X	X	X	X		X	X	X	X	X	X
Physical exam	X	X	X	X		X	X	X	X	X	X
Coartem® Dispersible given at Clinic	X	X	X	X							
Coartem® Dispersible given at home*	X		X								
Finger Prick to test for malarial infection	X	X	X	X	**X**	X	X	X	X	X	X
Blood Collected	X			X	X	X	X	X	X		X
Pregnancy test**	X										

- Sample informed consent (SIC), WHAT DOES MY CHILD HAVE TO DO IF HE/SHE IS IN THIS STUDY? section, sub-section Study Day 4: The DBS for malaria infection must be collected for all subjects. This section is modified as follows (the first clause in the 2nd bullet point has been deleted):

  At this visit the following will be done:
  - Your child will have about ½ teaspoon (3 mL) of blood drawn:
    - to measure the amount of Coartem® Dispersible in your child's blood.
  - **Y**our child will have his/her finger pricked to check for the amount of malarial parasites in your child's blood.

- Sample informed consent (SIC), WHAT DOES MY CHILD HAVE TO DO IF HE/SHE IS IN THIS STUDY? section, sub-section Study Days 14, 28, and 42: The addition of AL PK sample for study Day 14 and increase in estimate of total blood volume drawn. This section is modified as follows:

  At these visits the following will be done:
  - You will be asked questions about your child's symptoms, medications and medical history since the last visit.
  - Your child will have a short physical exam to assess any symptoms which are present.
  - Your child will have his/her finger pricked to check for the amount of malarial parasites in your child's blood.
  - Your child will have about ½ **to 1** teaspoon (2 – **5**.**4** mL) of blood drawn:
    - for routine blood tests,
    - to measure your child's HIV viral load (study Days 14 and 42 only),
    - to test for HIV resistance (study Day 42 only) **, and**
    - **to measure the amount of Coartem® Dispersible in your child's blood (Day 14 only).**

- IMPAACT P1079 Protocol Site Roster: Contact information for the Site Investigators is added. Kenneth Kintu replaces Monica Etima as the investigator for site 30293.

  - Site Investigator (12001)
    Portia Kamthunzi, MBBS, MTropaed, DTCH
    UNC Project
    Kamuzu Central Hospital
    Private Bag A-104
    Lilongwe, Malawi
    Phone: +265 999553034
    E-mail: portia85117@yahoo.co.uk; pkamthunzi@unclilongwe.org.mw

  - Site Investigator (30301)
    Macpherson Mallewa, B.Med.Sc, MBBS, MRCP, MRCPCH, DTMH, PhD
    College of Medicine
    Malawi-Liverpool-Wellcome Trust Clinical Research Programme
    P.O. Box 30096
    Chichiri
    Blantyre 3

Malawi
Phone: +265 1 876 444; +265 995835083
Email: mmallewa@mlw.medcol.mw; macphers@liverpool.ac.uk

- Site Investigator (30293)
Kenneth Kintu, MBchB, DPPM, MS
Makerere University-JHU Research Collaboration
Upper Mulago Hill Road
P.O. Box 23491
Kampala
Uganda
Phone: +256 414 541044/ +256 782 571450 (mobile)
E-mail: kkintu@mujhu.org

These updates will be made in the next version of the protocol when it is amended. Please contact the protocol team at actg.teamp1079@fstrf.org if you have any questions. Thank you for your interest in IMPAACT P1079.

Thank you for your participation in IMPAACT P1079.

APPENDIX I
SCHEDULE OF EVALUATIONS

	Scenario #1: Separate Screening and Entry Visits		OR	Scenario #2: Combined Screening and Entry Visits										
	Screening [1, 2]	Study Day 0 Entry[2,3]		Screening/ Study Day 0 Entry [1, 2,3]	Study Day 1[2]	Study Day 2[2]	Study Day 3	Study Day 4	Study Day 8 (+24 hour window)[4]	Study Day 14 (+24 hour window)[4]	Study Day 28	Study Day 42 End of study	Unscheduled Sick Visit	Early Discontinuation[20]
CLINICAL EVALUATIONS														
Informed Consent	X			X										
History[5]	X	X		X	X	X	X		X	X	X	X	X	X
Physical exam[6]	X	X		X	X	X	X		X	X	X	X	X	X
Adherence Assessment[7]	X	X		X	X	X	X							
Artemether-lumefantrine (AL) dosing[8]		Dose 1- clinic Dose 2- home		Dose 1- clinic Dose 2- home	Dose 3- clinic	Dose 4- clinic Dose 5-home	Dose 6- clinic[9]							
LABORATORY EVALUATIONS														
Hematology[10]	1 mL			1 mL						1 mL	1 mL	1 mL		1 mL
Chemistries[11]	1 mL			1 mL						1 mL	1 mL	1 mL		1 mL
Thick Blood Smear (finger stick)	0.05	0.05		0.05	0.05	0.05	0.05		0.05	0.05	0.05	0.05	0.05	0.05
Thin Blood Smear (finger stick)	0.05			0.05										
Dried Blood Spots (DBS) for Malaria Infection[12]		0.1 mL		0.1 mL	0.1 mL	0.1 mL	0.1 mL	0.1 mL	0.1 mL	0.1 mL	0.1 mL	0.1 mL	0.1 mL	0.1 mL
Urine [or Blood] Pregnancy test [13]	2 mL [1-2 mL] [17]	2 mL[16] [1-2 mL] [17]		2 mL [1-2 mL] [17]										
Virology														
DBS for Viral Load[14]	0.25 mL	0.25 mL[16]		0.25 mL					0.25 mL	0.25 mL		0.25 mL		0.25 mL
Drug Resistance Testing (DBS)[15]	0.25 mL	0.25 mL[16]		0.25 mL								0.25 mL		0.25 mL
Pharmacology														
Artemether-lumefantrine (AL) PK[18]							15 mL	3 mL	3 mL	3 mL				
Nevirapine PK[19]		1 mL		1 mL			1 mL							
TOTAL BLOOD VOLUMES	2.6-4.6 mL	1.15-3.65 mL		3.7-5.7 mL	0.15 mL	0.15 mL	16.15 mL	3.1mL	3.4 mL	5.4 mL	2.15 mL	2.65 mL	0.15 mL	2.65 mL

APPENDIX I (Cont.)

1. Screening evaluations must be performed within 30 days of Entry.
2. Note that screening and entry can occur on the same day. In addition, for subjects who have started AL prior to study consent and have received up to 3 doses of AL, study Days 0, 1 and 2 evaluations may all be collapsed into a single visit, consistent with all criteria listed on study Day 0 (entry visit). Subjects who enter the study after having initiated AL may have a study Day 0 visit, with the next visit matched to the subject's day of AL treatment.
3. Subjects with Grade 3 or 4 hemoglobin at entry are allowed to participate in all study procedures (e.g. evaluation of the 28 and 42-day safety and toxicity) EXCEPT for the AL PK sampling or single samples for NVP measurements (to mimimize the volume of blood drawn in these children).
4. Team should be notified within 24 hours if timelines (i.e. +24 window) can not be met.
5. A complete history is required at Screening/Entry Visit; a targeted history [i.e. review of new illnesses and/or symptoms] is sufficient at subsequent visits.
6. Complete physical exam (including height, weight, vital signs [temperature, pulse, respiration, and blood pressure]) at Screening/Entry Visit and targeted physical exam (including vital signs [temperature, pulse, respiration, and blood pressure]) is sufficient at all other visits.
7. Adherence assessment will be conducted both retrospectively for the doses AL already taken, as well as, prospectively. All doses require documentation of date and time.
8. Dosing of AL must be performed according to schedule included in Section 5.1.
9. Draw Pre-Dose PK sample, then administer Dose 6 (the last dose) of AL.
10. Hematology should include: CBC.
11. Chemistries should include: AST, ALT, and serum creatinine.
12. See section 6.214. Prepare 1 card with 2 spots at 0.05 mL each spot. Batched and shipped according to the instructions provided in the Laboratory Processing Chart (LPC). Note: DBS blood can be collected in a vacutainer tube ONLY when blood is collected for other laboratory evaluations (i.e. hematology, chemistry, or pharmacokinetic). All other DBS collections should be performed via finger stick. See Appendix III for additional collection, processing, and storage instructions.
13. Must be performed within 72 hours of enrollment on female subjects who have had their first menstrual period.
14. Prepare 1 card with 5 spots at 0.05 mL each spot. Batched and must be performed at DAIDS VQA-certified laboratory. Note: DBS blood can be collected in a vacutainer tube ONLY when blood is collected for other laboratory evaluations (i.e. hematology, chemistry, or pharmacokinetic). All other DBS collections should be performed via finger stick. See Appendix III for additional collection, processing, and storage instructions.
15. Prepare 1 card with 5 spots at 0.05 mL each spot. Collect on all study participants. To be performed on subjects who show changes in viral load pattern between study Day 0 and study Day 42 using dried blood spot collected at screening and study Day 42. Note: DBS blood can be collected in a vacutainer tube ONLY when blood is collected for other laboratory evaluations (i.e. hematology, chemistry, or pharmacokinetic). All other DBS collections should be performed via finger stick. See Appendix III for additional collection, processing, and storage instructions.
16. This evaluation should only be performed if it was not performed at Screening.
17. Blood pregnancy test can be substituted for urine pregnancy test.
18. AL pharmacokinetics (3 mL per sample) will be collected on study Day 3: Pre-dose, 1, 2, 4, and 8 hours; study Day 4: 24 hours; study Day 8: 120 hours post-administration of the last AL dose; and study Day 14: 264 hours post-administration of the last AL dose. Participants will be asked to remain within the clinic or research facility until completion of the 8 hour blood sample collection at which time they will be discharged for home. They will be asked to return to the clinic or research facility for the 24 hour (study Day 4) and 120 hour (study Day 8) blood sample collections. For subjects who experience treatment failure before study Day 3, PK sampling will be discontinued. Specimen aliquots are prepared according to instructions provided in the LPC.
19. NVP pharmacokinetic samples will be collected on study Day 0 and study Day 3. The baseline (study Day 0) pharmacokinetic samples of NVP will be collected at random times prior to initiating AL treatment and following the previous NVP dose (approximately 12 hours following the evening dose of NVP). The second sample, study Day 3, will be collected just prior to the morning NVP dose and at the time of the last dose of AL. Specimen aliquots are prepared according to instructions provided in the LPC.
20. Subjects who prematurely discontinue study treatment will continue to be followed at scheduled study visits, but only the evaluations listed in this column are required.

For insufficient blood draws at SCREENING visit, priorities are as follows:

a. Hematology
b. Chemistries
c. Nevirapine PK

For insufficient blood draws at DAY 3 visit, priorities are as follows:

a. Artemether-lumefantrine (AL) PK
b. Nevirapine PK

To: IMPAACT Principal Investigators and Study Coordinators at Sites Participating in IMPAACT P1079

From: IMPAACT P1079 Protocol Team

Date: June 15, 2010

Re: Clarification Memo #1 for Protocol P1079, Version 1.0, dated May 21, 2010, entitled "Pharmacology of Artemisinin-Based Antimalarial Therapy Within The Context Of Antiretroviral Therapy"

This is Clarification Memo #1 for IMPAACT P1079, Version 1.0, dated May 21, 2010. This Memo can be obtained from the P1079 protocol-specific web page (http://www.impaactgroup.org/p1079-protocol-specific-web-page). The username is *impaact* and the password is *cure* (all lower case). The document is located under the section titled Current Protocol Related Documents.

This memo serves to change the word 'DRAFT' to 'FINAL' on page 1 of the sample informed consent (SIC).

This clarification will be included in the next version of the protocol when it is amended. Please contact the protocol team at actg.teamp1079@fstrf.org if you have any questions. Thank you for your interest in IMPAACT P1079.

Sincerely,

The P1079 Protocol Team

IMPAACT P1079

PHARMACOLOGY OF ARTEMISININ-BASED ANTIMALARIAL THERAPY WITHIN THE CONTEXT OF ANTIRETROVIRAL THERAPY

Multicenter, International Trial of the
International Maternal Pediatric Adolescent AIDS
Clinical Trials Group (IMPAACT)

Sponsored by:

The National Institute of Allergy and Infectious Diseases (NIAID)

and

The Eunice Kennedy Shriver National Institute of Child Health and Human Development (NICHD)

Non-IND Protocol

IMPAACT Complications Scientific Committee Chair: Sharon Nachman, M.D.

Protocol Chair:	Francesca Aweeka, Pharm.D.
Protocol Co-Vice Chairs:	Sunil Parikh, M.D., M.P.H.<br>Sharon Nachman, M.D.
NIAID Medical Officer:	Paul Sato, M.D., M.P.H.
NICHD Medical Officer:	George K. Siberry, M.D.
Clinical Trials Specialist:	Kimberly R. Hudgens, M.S.H.C.A., M.B.A.

Version 1.0
FINAL
May 21, 2010

IMPAACT P1079 PROTOCOL TEAM ROSTER

All questions concerning this protocol should be sent via e-mail to actg.teamp1079@fstrf.org. Remember to include the subject's PID when applicable. The appropriate team member will respond to questions via e-mail with a "cc" to actg.teamp1079@fstrf.org. A response should generally be received within 24 hours (Monday - Friday). For protocol registration questions, e-mail protocol@tech-res.com or call 301-897-1707. Protocol registration material can be sent electronically to epr@tech-res.com or via fax at 1-800-418-3544 or 301-897-1701. For EAE questions, e-mail DAIDSRSCSafetyOffice@tech-res.com or call 1-800-537-9979 or 301-897-1709, or fax 1-800-275-7619 or 301-897-1701. For randomization or enrollment questions, contact the Data Management Center at 716-834-0900 or by email at sdac.random.desk@fstrf.org .

Protocol Chair & Pharmacologist

Francesca Aweeka, Pharm.D.
Department of Clinical Pharmacy
University of California, San Francisco
San Francisco, CA 94143
Phone: (415) 476-0339
Fax: (415) 476-0307
E-mail: faweeka@sfghsom.ucsf.edu

Protocol Co-Vice Chairs

Sunil Parikh, M.D., M.P.H.
Division of Infectious Diseases
Department of Medicine
University of California, San Francisco
San Francisco, CA 94143
Phone: (415) 206-8687
E-mail: sparikh@medsfgh.ucsf.edu

Sharon Nachman, M.D.
Department of Pediatrics
SUNY Stony Brook
HSC T11-031
Stony Brook, NY 11794
Phone: (631) 444-7692
E-mail: Sharon.Nachman@stonybrook.edu

NIAID Medical Officer

Paul Sato, M.D., M.P.H.
TRP/IMAPB
DAIDS, NIAID
6700 B Rockledge Drive, Room 5218
Bethesda, MD 20817
Phone: (301) 435-3750
E-mail: satop@niaid.nih.gov

NICHD Medical Officer

George K. Siberry, M.D., M.P.H.
Ped Adol Maternal AIDS Branch
Eunice Kennedy Shriver National Institute of Child Health and Human Development
6100 Executive Boulevard, Room 4B11H
Rockville, MD 20852
Phone: (301) 496-7350
Fax: (301) 496-8678
E-mail: siberryg@mail.nih.gov

Clinical Trials Specialist

Kimberly R. Hudgens, M.S.H.C.A., M.B.A.
IMPAACT Operations Center
Social & Scientific Systems, Inc.
8757 Georgia Avenue, 12th Floor
Silver Spring, MD 20910
Phone: (301) 628-3349
Fax: (301) 628-3304
E-mail: khudgens@s-3.com

IMPAACT P1079 PROTOCOL TEAM ROSTER

Protocol Statisticians

Vincent J. Carey, Ph.D.
Senior Statistician, Pediatric Division
Statistical & Data Analysis Center
Harvard School of Public Health
Building 3-505C, Channing Laboratory
677 Huntington Avenue
Boston, MA 02115-5821
Phone: (617) 525-2265
Fax : (617) 731-1541
E-mail : stvjc@channing.harvard.edu

Mona Farhad, M.S.
Statistical & Data Analysis Center
Harvard School of Public Health
FXB Building, Room 501
651 Huntington Avenue
Boston, MA 02115-6017
Phone: (617) 432-7524
Fax: (617) 432-3163
E-mail: mfarhad@sdac.harvard.edu

Protocol Data Manager

Bobbie Graham, B.S.
Frontier Science & Technology
Research Foundation
4033 Maple Road
Buffalo, NY 14226-1056
Phone: (716) 834-0900 x7265
Fax: (716) 834-8675
E-mail: graham.bobbie@fstrf.org

Field Representatives

Maureen M. Haak, R.N., M.S.N
Research Coordinator
Rush University Cook County Hospital
2020 W. Harrison, Room 2130
Chicago, IL 60612-7324
Phone: (312) 572-4541
Fax: (312) 572-4559
E-mail: maureen_haak@rush.edu

Carol A. Vincent, C.R.N.P., M.S.N
Project Manager/Study Coordinator
The Children's Hospital of Philadelphia
Allergy and Immunology
3535 Market Street
11th Floor, Suite 1008, Room 1109
Philadelphia, PA 19104-4318
Phone: (215) 590-2262
Fax: (215) 590-5653
E-mail: vincentc@email.chop.edu

Protocol Pharmacist

Lynette Purdue, Pharm.D.
NIH, NIAID, DAIDS, PAB
ROOM 4107
6700-B Rockledge Drive MSC 7620
Bethesda, MD 20892-7620
Phone: (301) 435-3744
Fax: (301) 402-1506
E-mail: LPURDUE@niaid.nih.gov

Protocol Virologist

Deborah Persaud, M.D.
Johns Hopkins School of Medicine
Pediatric Infectious Diseases
200 North Wolfe Street Room 3151
Baltimore, MD 21205
Phone: (443) 287-3733
Fax: (410) 955-0964
E-mail: dpers@jhmi.edu

Protocol Immunologist

Savita G. Pahwa, M.D.
University of Miami School of Medicine
Batchelor Research Institute, Room 712
1580 Northwest 10th Avenue
Miami, FL 33136
Phone: (305) 243-7732
Fax: (305) 243-7211
E-mail: spahwa@med.miami.edu

IMPAACT P1079 PROTOCOL TEAM ROSTER

Laboratory Technologist

Tim Cressey, B.Sc., Ph.D.
Program for HIV Prevention and Treatment (PHPT)/IRD 174
Faculty of Associated Medical Sciences
Department of Clinical Microbiology
110 Inthawaroros Road
Muang Chiang Mai 50200
Thailand
Phone: 66 (0) 53 894 431
Fax: 66 (0) 53 894 220
E-mail: tim@phpt.org

Laboratory Data Coordinator

Travis Behm, B.S.
Frontier Science and Technology Research Foundation
4033 Maple Road
Amherst, NY 14226
Phone: (716) 834-0900 x7377
Fax: (716) 833-0655
E-mail: tbehm@fstrf.org

Investigators

Omolara Aderinboye, M.D.
Department of Pediatrics
SUNY Stony Brook
HSC T11-031
Stony Brook, NY 11794-8111
Phone: (631) 444-7692
E-mail:
Omolara.Aderinboye@stonybrook.edu

Investigators (cont.)

Charlotte Hobbs, M.D.
Department of Pediatrics
Division of Infectious Diseases
NYU School of Medicine
550 First Avenue
New York, NY 10016
Phone: (212) 263-6426
Fax: (212) 263-7806
E-mail: hobbsc01@med.nyu.edu

Werner Schimana, M.D.
Consultant Pediatrician
Kilimanjaro Christian Medical Center
Pediatrics
P.O. Box 3010
Moshi, Tanzania
Phone: (255) 0754-780220
E-mail: wschimana@pedaids.org

Community Advisory Board Representative

Teopista Nakyanzi
Makerere University and Johns Hopkins University Research Collaboration
Old Mulago Hill Road
Kampala, Uganda
Phone: (256) 753 496913
E-mail: tnakyanzi@yahoo.com

IMPAACT P1079 PROTOCOL SITE ROSTER

Site 12001- University of North Carolina Lilongwe CRS
Kamuzu Central Hospital/Tidziwe Centre
Mzimba Road
Lilongwe
Malawi

CRS Leader: Francis Martinson, MPH, PhD, MBCHB
Phone: 265-8838388
Email: fmartinson@unclilongwe.org.mw
CRS Coordinator: Mina C. Hosseinipour, MD
Phone: 265-1758938
Email: minach@med.unc.edu

Site 30293- Makerere University- JHU Research Collaboration [MUJHU CARE LTD] CRS
Upper Mulago Hill Road
MUJHU Research House
Kampala
Uganda

CRS Leader: Philippa Musoke, MBCHB
Phone: 256-41-541044
Email: pmusoke@mujhu.org

CRS Investigator: Monica Etima, MBCHB
Email: metima@mujhu.org

ICAB Rep: Teopista Nakyanzi
Phone: 256-753-496913
Email: tnakyanzi@yahoo.com

Site 30301- College of Med. JHU CRS
Queen Elizabeth Central Hospital
Chipatala Avenue
Blantyre
Malawi

CRS Leader: Newton Kumwenda, MPH, PhD
Phone: 265-1875129
Email: nkumwenda@jhu.medcol.mw
CRS Coordinator:
Leslie Degnan, MPH
Phone: 265-888-208609
Email: ldegnan@jhu.medcol.mw

GLOSSARY OF COMMONLY USED DEFINITIONS AND ABBREVIATIONS

ACT	artemisinin-based combination therapy
AE	Adverse Event
AL	artemether/lumefantrine
AQ/AS	amodiaquine/artesunate
AR	artemether
ARV	antiretroviral
ART	Antiretroviral therapy
BSA	Body surface area
CAP	College of American Pathologists
CLIA	Clinical Laboratory Improvement Amendments
CRF	Case report form
CV	Coefficient of Variation
CYP	cytochrome p450
DAERS	DAIDS Adverse Event Reporting System
DHA	dihydroartmesinin
DP	dihydroartemesinin/piperaquine
EAE	Expedited Adverse Event
ECG	electrocardiogram
EFV	efavirenz
FDA	Food and Drug Administration
HAART	highly active antiretroviral therapy
Hb	hemoglobin
ICH	International Conference on Harmonization
LPV/r	lopinavir/ritonavir
NNRTI	non-nucleoside reverse transcriptase inhibitor
NRTI	nucleoside reverse transcriptase inhibitor
NVP	nevirapine
PI	Protease inhibitor
PK	pharmacokinetic
RSC	Regulatory Support Center
SAE	serious adverse event
SP	sulfadoxine-pyrimethamine
TNF-α	tumor necrosis factor-alpha
UGT	UDP-glucuronosyltransferases
VQA	Virology Quality Assurance Program
WHO	World Health Organization

TABLE OF CONTENTS

GLOSSARY OF COMMONLY USED DEFINITIONS AND ABBREVIATIONS 5

SCHEMA 8

1.0 INTRODUCTION 10
1.1 Background 10
1.2 Rationale 14

2.0 STUDY OBJECTIVES 16
2.1 Primary Objectives 16
2.2 Secondary Objectives 16

3.0 STUDY DESIGN 17
3.1 Overview 17
3.2 AL Dosing and Pharmacokinetics 18
3.3 NVP-Based ARV Therapy and Pharmacokinetics 19

4.0 SELECTION AND ENROLLMENT OF SUBJECTS 19
4.1 Inclusion Criteria 19
4.2 Exclusion Criteria 21
4.3 Disallowed Medication Guidelines 22
4.4 Enrollment Procedures 23
4.5 Co-enrollment Procedures 24

5.0 STUDY TREATMENT 24
5.1 Drug Regimens, Administration and Duration 24
5.2 Drug Formulation 27
5.3 Drug Supply, Distribution and Pharmacy 27

6.0 SUBJECT MANAGEMENT 28
6.1 Toxicity Management 28
6.2 Study Management Plan 29
6.3 Criteria for Early/Late Treatment Failure 31
6.4 Criteria for Study Drug (AL) Discontinuation 32
6.5 Criteria for Study Discontinuation 32

7.0 ADVERSE EVENT REPORTING 33
7.1 Adverse Event Reporting to DAIDS 33
7.2 Reporting Requirements for this Study 33
7.3 Grading Severity of Events 33
7.4 Expedited AE Reporting Period 33

8.0 STATISTICAL CONSIDERATIONS 34

8.1 General Design Issues........34
8.2 Outcome Measures........34
8.3 Randomization and Stratification........34
8.4 Sample Size and Accrual........35
8.5 Monitoring........36
8.6 Analyses........37

9.0 CLINICAL PHARMACOLOGY PLAN........38
9.1 Pharmacology Objectives........38
9.2 Study Design, Modeling and Data Analysis........38
9.3 Virologic Sampling........40

10.0 HUMAN SUBJECTS........40
10.1 Institutional Review Board and Informed Consent........40
10.2 Subject Confidentiality........41
10.3 Study Discontinuation........41

11.0 PUBLICATION OF RESEARCH FINDINGS........41

12.0 BIOHAZARD CONTAINMENT........41

13.0 REFERENCES........42

<u>APPENDICES:</u>

I. SCHEDULE OF EVALUATIONS

II. BLOOD VOLUME COLLECTION IN SMALL CHILDREN

III. DRIED BLOOD SPOT COLLECTION, PROCESSING AND STORAGE INSTRUCTIONS

IV. GUIDANCE FOR DETERMINING SEVERE MALNUTRITION

V. COARTEM® DISPERSIBLE DOSAGE AND ADMINISTRATION DIAGRAM

VI. IMPAACT SAMPLE INFORMED CONSENT TEMPLATE

SCHEMA

Pharmacology of Artemisinin-Based Antimalarial Therapy Within the Context of Antiretroviral Therapy

DESIGN: Prospective, pharmacokinetic (PK) study to investigate the drug-drug interaction between the antimalarial treatment artemether/lumefantrine (AL) and nevirapine (NVP)-based antiretroviral (ARV) treatment for HIV, in co-infected children in resource limited settings.

SAMPLE SIZE: 48 evaluable HIV-infected children; 24 subjects per study arm.

POPULATION: Two study arms will include children who are either on NVP-based ARV treatment or no ARV treatment at the time of presenting with non-severe malaria* to sites that routinely treat pediatric malaria with AL:

- Arm A (n=24): NVP-based ARV treatment (ages ≥3 to ≤12 years)
- Arm B (n=24): No ARV treatment (ages ≥3 to ≤ 12 years)

Note: Subjects must be receiving (a) NO ARV drugs for at least 4 weeks prior to study entry, OR (b) NVP-based combination ARV therapy for at least 4 weeks prior to study entry. Children receiving NO ARV drugs are not yet on ARVs because they do not meet national guidelines for needing such therapy.

* See Section 4.27 for definition of severe malaria.

AGE DISTRIBUTION: The objective is to achieve approximate balance of age distribution between arms in proportions of subjects above and below six years of age.

REGIMEN:

- Children who test positive for malaria will be given a total of 6 doses of AL, distributed over study Day 0 to study Day 3*. Specifically, children will receive 2 weight-based doses of AL, approximately 8-12 hours apart on Study Day 0 (study entry), and on study Day 2 of the study.
- On study Days 1 and 3, subjects will be given AL once daily (See Section 5.1, Table 1).
- Subjects will also continue to receive the same NVP- based treatment prescribed by their physician (if any) for their HIV

infection that they were receiving at the time of malaria presentation.

*Note: For subjects who have started AL prior to study consent and have received up to 3 doses of AL, study Days 0, 1 and 2 evaluations may all be collapsed into a single visit, consistent with all criteria listed on study Day 0 (entry visit). Subjects who enter the study after having initiated AL may have a study Day 0 visit, with the next visit matched to the subject's day of AL treatment.

TREATMENT DURATION: 6 doses of AL over a 4 day period (Study Day 0 to Study Day 3).

STUDY DURATION: Each subject will be followed for 42 days from entry. The study is expected to accrue over a 1-year period.

OBJECTIVES:

Primary:

1. To determine if NVP-based ARV therapy versus no ARV therapy alters the AL PK exposure in children (ages ≥3 to ≤12 years) who are co-infected with malaria and HIV.

2. To evaluate the 28 and 42-day safety and toxicity following co-administration of 6 doses of AL with NVP. (Arm A only).

Secondary:

1. To determine if HIV infection is associated with altered disposition of AL.

2. To explore the effects of malaria co-infection and artemisinin based treatment on HIV viral load in children who are and are not receiving ARV.

3. To explore the effects of malaria co-infection on selection of ARV drug resistance among children receiving NVP-based HAART and experiencing increase in viral loads.

4. To investigate the impact of AL on NVP pharmacokinetics.

5. To characterize the frequency and factors associated with early and late treatment failures.

1.0 INTRODUCTION

1.1 Background

Malaria is endemic throughout the tropical areas of the world and is acquired from the bite of the female Anopheles species mosquito. Worldwide, there are 300 to 500 million cases annually and 1.5 to 2.7 million deaths.(1) Most deaths occur in young children in regions of sub-Saharan Africa. Malarial infection poses substantial risks to pregnant women and their fetuses and may result in spontaneous abortion and stillbirth. In addition, co-infection with HIV complicates malaria therapy as patients receive ARVs concurrently, which may lead to significant drug-drug interactions.

The diagnosis of malaria is based on clinical criteria such as fever supplemented by the detection of parasites in the blood as described in the 2006 guidelines of the World Health Organization (WHO). Due to increasing *Plasmodium falciparum* resistance to older drugs, treatment has expanded from affordable chloroquine and sulfadoxine-pyrimethamine (SP) to more costly artemisinin-based combination therapy (ACT). In 2001, the WHO recommended the use of ACTs for the treatment of uncomplicated malaria. Since then, the majority of *falciparum* endemic countries have adopted the ACTs as first-line treatment.(2) ACT includes an artemisinin drug with a short half-life that rapidly diminishes parasite burden and fever plus a drug with sustained activity. Initially, the ACT regimen of amodiaquine/artesunate (AQ/AS) was considered to be among the most appropriate regimens to adopt in Africa. However, its use is moving toward disfavor due to unexpected toxicities in the setting of HIV disease(3) and is being replaced by other ACTs, such as artemether-lumefantrine (AL) and dihydroartemesinin/piperaquine (DP). In fact, the most commonly adopted ACT regimen in Africa is AL. As of September 2007, 37 countries in the world have adopted AL as their first line antimalarial therapy, including 25 countries in Africa (http://malaria.who.int/treatmentpolicies.html). Despite the widespread adoption and deployment of ACT regimens for the treatment of millions of malaria infections per year, our knowledge of their pharmacokinetics remains inadequate, especially in children. For the purposes of this study, we will focus on the pharmacokinetics of AL in HIV-malaria co-infected children treated with an NVP-based ARV regimen. The brand of AL that will be used for this study is Coartem® Dispersible (20 mg of artemether and 120 mg of lumefantrine), manufactured by Novartis.

AL Efficacy in Children:

AL is highly efficacious for treatment of uncomplicated malaria. However, due to a less-developed immunity, children have both higher rates of infection and higher rates of treatment failure (recrudescence) and new infection compared to adults.(4) In a recent study of 403 children in Uganda (ages 1-10 years), treatment with AL resulted in a high new infection rate (confirmed by fingerprinting of parasites) of 27%, (5) data comparable to a Tanzanian study(6). Distinct pharmacologic characteristics in children (i.e. enhanced drug metabolism) in addition to immunologic distinctions, may in part explain the higher rates of recrudescence and re-infection in children.

AL Pharmacology and Exposure-Response:

AL pharmacology is highly complex.(7) Artemether is rapidly demethylated to active dihydroartmesinin (DHA) by cytochrome P450 (CYP) 3A4/5 which undergoes further metabolism via UDP-glucuronosyltransferases (UGT).(8) Artemether induces CYP2C19 and CYP3A4.(9) Lumefantrine is metabolized via CYP3A4 and inhibits CYP2D6.(10) Factors affecting AL pharmacokinetic (PK) exposure may include CYP/UGT maturation in children and drug-drug interactions. Lumefantrine PK exposure is also affected by whether or not a high-fat meal is consumed at the time of drug administration. Bioavailability of lumefantrine and artemether increased 16 and 2-fold, respectively, in healthy adults consuming a high-fat meal compared to when fasting.(7;11)

Grapefruit juice significantly increases the oral bioavailability of artemether without an effect on the elimination half-life through metabolism via the CYP3A4 pathway, which it inhibits. However, grapefruit juice is not routinely part of the diet for children in the countries that this study will be conducted (Uganda and Malawi). While it is possible that grapefruits will be available in country, the team recommends that attention be paid to exclusion of grapefruit and grapefruit juice from the child's diet during the time of administration of the Coartem® Dispersible (20 mg of artemether and 120 mg of lumefantrine).

Pediatric PK data on AL are limited. Doses for children are generally deduced from adult doses adjusted for body surface area, probably resulting in suboptimum PK for many drugs(12-14) as CYP/UGT metabolism is higher in pre-pubescent children.(15) We hypothesize that AL metabolism is higher in children than adults, resulting in lower overall PK exposure, thereby increasing the risk for treatment failure. To assess the impact of age on AL pharmacokinetics, Ugandan children aged 5-13 years with uncomplicated malaria (n=20) were recently evaluated (16). Comparison to prior adult data suggests that lumefantrine exposure is lower in children. Results for artemether(AR)/DHA revealed that mean $C_{\mathbf{max}}$ and $AUC_{\mathbf{0}-\infty}$ values for AR were approximately 2 to 3-fold higher in children compared to adults. Exposure to DHA was similarly higher in children than

healthy adults ($AUC_{0-\infty}$ 382 vs. 198 h·ng/ml). It should be noted, however, that comparing PK data generated in malaria-infected patients to data generated in healthy adults is a limitation in that acute malaria infection alone may impact the disposition of artemisinin drugs. Thus, the current proposal will enroll malaria-infected children as controls.

CYP/UGT activity varies with age. Several clinical pharmacokinetic studies have reported higher clearance in prepubescent children as compared to adults. Cyclosporine clearance via CYP3A4 was shown to correlate more closely with BSA rather than body weight and was higher in children <8 years of age compared to adults.(13) Clearance of UGT substrates (e.g. morphine) has been reported to be greater than body-weight normalized clearance.(15)

Pharmacodynamic studies of ACT regimens have focused mostly on the longer-acting lumefantrine. A relationship between lumefantrine exposure and therapeutic response was observed in Mbarara, Uganda. Re-infections, in a population of mostly children, were more prevalent when lumefantrine concentrations below 400 ng/mL were measured 7 days from the start of treatment (6 dose regimen) ($p < 0.001$).(17) Likewise, in a Thai population, patients who had lumefantrine levels below 175 ng/mL on day 7 were more likely to experience recrudescence by day 42 (adjusted hazard ratio, 17; 95% CI, 5.5-53), allowing prediction of treatment failure with 75% sensitivity and 84% specificity.(18) Thus, factors reducing lumefantrine exposure (e.g. drugs inducing CYP3A4 metabolism such as NVP) may compromise treatment.

AL Toxicity:

The artemisinins overall are generally well tolerated in humans. In a clinical safety review of 108 studies including 9241 patients, no serious adverse events or significant toxicities were reported.(19) Of minimal concern is the potential for artemisinin associated auditory toxicity. However, in a study based in Thailand, no evidence of auditory toxicity was reported.(20) Longer acting lumefantrine is also generally well tolerated. The drug is chemically similar to halofantrine, a drug associated with cardiac arrhythmogenic potential, but no cardiac issues have been identified in children receiving lumefantrine. In 713 lumefantrine treated patients, serial ECG monitoring indicated no adverse cardiac events.(21) There have been no reports associating elevated AL drug levels with toxicity.

In contrast to the safety profile of AL and to provide an explanation for its increasing use, there is growing concern over the toxicity of AQ/AS, especially in HIV co-infected patients. AQ was previously associated with hepatitis when used for prolonged periods resulting in discontinued use as a prophylactic agent. The mechanism of toxicity is presumed to be associated with toxic metabolites. Our group recently reported unexpected hepatitis in two of two healthy volunteers receiving AQ/AS and efavirenz (EFV) concomitantly with each subject exhibiting

increased AQ exposure (as measured by AUC, 115 and 302 percent, respectively).[22] In a study in children treated for malaria in Kampala, neutropenia was significantly more common in an HIV-infected cohort compared to an HIV-uninfected cohort receiving amodiaquine (45% vs. 6% respectively) with the risk higher in those taking antiretrovirals compared to those not taking antiretrovirals (75% vs. 26%).[3]

HIV and Malaria Co-Infection and Drug-Drug Interactions:

Malaria and HIV are two of the most common infections in sub-Saharan Africa and, to a lesser extent, in other developing countries. According to the UNAIDS, at the end of 2007, 22.3 million persons were living with HIV in sub-Saharan Africa and there were 33.2 million HIV infected persons worldwide[23] whereas 300 million to 500 million suffer from malaria each year.[24] Clinically significant drug-drug interactions between ACT and ARV are expected. In addition to PK effects, HIV infection itself may alter response to ACT, as differences in ACT activity have been reported between HIV-infected and HIV-uninfected adults.[25]

Interestingly, due to a theoretical PK interaction but in the absence of any data, AL was previously avoided in many parts of sub-Saharan Africa when ARV co-administration was warranted. However, with recent reports in HIV-infected patients of unexpected toxicity with AQ/AS, AL use is again on the rise in this population, underscoring the need to evaluate these potential PK interactions. The primary concern is the impact of ARV on AL PK, as any PK alteration to AL may alter the antimalarial efficacy of AL. Indeed, NVP or EFV based ARV regimens are well known to affect PK of CYP substrates, and it is expected that NVP will induce the metabolism of AL resulting in diminished exposure of both artemether and lumefantrine, compromising efficacy. Conversely, given that the AL treatment is only for three days, there is little concern of an impact of AL exposure on ARV efficacy.

To date, German et al., has evaluated the interaction between AL and lopinavir/ritonavir (LPV/r) in healthy adult volunteers and reported a 200 percent increase in lumefantrine exposure with LPV/r co-administration [26]. In spite of the increase in exposure, the safety profile of lumefantrine suggests that dosage adjustment may not be necessary. No data are available to date investigating the interaction between AL and NVP which will be the focus of this study. NVP may diminish lumefantrine exposure, due to CY3A4 induction, which may compromise the effectiveness of the 3 day regimen. Furthermore, no data are available for any of these interactions in children.

Currently, the WHO recommends the use of two nucleoside reverse transcriptase inhibitors (NRTIs) plus one non-nucleoside reverse transcriptase inhibitor (NNRTI) as the first-line regimen for the treatment of HIV infection in infants

and children living in resource-limited settings, the majority of whom live in Africa and Asia where the proposed studies are planned. NVP is a commonly prescribed NNRTI for an HIV-infected cohort in Kampala, Uganda and is commonly used at other IMPAACT international sites. Several recent trials have supported the use of co-trimoxazole in most HIV-infected individuals in sub-Saharan Africa.[27] Benefits include reductions in all-cause morbidity and mortality, with specific reductions in malaria and bacterial pneumonia. It is therefore expected that the study participants will also be receiving co-trimoxazole. We do not, however, expect an effect of co-administration of co-trimoxazole on the pharmacokinetics of AL, the regimen to be studied in this protocol.

Potential Impact of Malaria and ACT on HIV Viral Load:

The effect of malaria co-infection on plasma viral loads in children has not been well-characterized. Studies in HIV-infected adults have shown that co-infection with malaria results in an increase (doubling or 0.25-log increase) in viral load levels relative to steady-state (pre-malaria infection) levels. The increase in plasma viral load levels observed during malaria co-infection in adults was greater (mean 0.82-log relative to pre-malaria infection levels) among individuals with high levels of CD4+ T cells (>300 cells/µL), and high parasite density (≥2000/ µL).[28;29] In general, in adults, a return of plasma viral load levels to baseline following malaria infection was found to occur by 8-9 weeks post-infection. The sustained increase in viral loads following malaria co-infection is attributed to sustained increases in pro-inflammatory cytokines such as Tumor Necrosis Factor-alpha (TNF-α) in co-infected individuals.[30] Interestingly, the anti-malaria drug artesunate has been shown *in vitro* to have antiviral activity against cytomegalovirus, hepatitis B and C virus; the mechanism of which is unknown, and a possible effect on HIV-1 has been implicated.[31-35] In this study which examines the interaction between ACT and NVP-based ARV in HIV-1 infected children with malaria, some of whom will be receiving ARV therapy, we will explore the changes in HIV-1 viral loads during malaria co-infection and during treatment with ACT. If increases in plasma viremia are observed over sequential time points among the children receiving NVP-based HAART, we will also examine whether the increase is associated with selection of NVP drug resistance.

1.2 <u>Rationale</u>

ACTs are the mainstay of antimalarial therapy throughout much of the world, yet pediatric PK data on the most widely adopted ACT regimen, AL, are lacking and were the focus of recent work carried out in Kampala, Uganda. Children exhibit maturation in metabolic processes which may result in lower drug exposure of the ACTs compared to adults. Of equal importance is the assessment of key drug-drug interactions in HIV co-infected children as ARVs are known to affect the

metabolic enzyme activity responsible for ACT elimination. Data presented at the International AIDS Society meeting in Sydney, Australia, indicates HIV infected children benefit from early versus deferred ARV therapy, which has resulted in increased use of ARVs and increased likelihood of ARV and antimalarial concomitant therapy, particularly in the populations most vulnerable to failure of AL therapy.

Malaria and HIV are among the two most important global health problems of our time. Together, they cause more than four million deaths per year. Malaria accounts for more than a million deaths each year, of which 90% occur in Africa where malaria is the leading cause of mortality in young children. Sub-Saharan Africa is also home to more than 29 million people living with HIV/AIDS. In 2003 in Africa, AIDS claimed the lives of an estimated 2.4 million people and over 600,000 children were newly infected with the virus. The incidence of malaria according to WHO world malaria report 2005 by our countries of interest is as follows: Uganda 477.9/1000, Tanzania 289.7, Malawi 240.4, Zambia 190.2, Zimbabwe 97.6, Nigeria 21, Botswana 12.6, Kenya 3.9, India 1.7 and Thailand 0.6. The prevalence of HIV in many of these countries is above 6% in children younger than 15 years.

Of primary concern is the impact of ARV therapy on AL drug exposure in HIV-malaria co-infected children, since the latter is administered for three days and increases or decreases in its exposure may predispose children to AL toxicity or treatment failure, respectively. AL pharmacology is complex, so the magnitude of an interaction must consider the PK of both the parent drugs and their active metabolites. AL is the most common ACT regimen in current use. Since there is evidence that diminished lumefantrine may compromise outcomes, we will prioritize the potential interaction between the CYP 3A4 inducer, NVP, and AL.

Nevirapine is more widely prescribed for children in the developing world than any other NNRTI or protease inhibitor (PI). It is often administered as part of a fixed dose combination regimen including NVP with lamivudine and stavudine (Triomune®) or NVP with lamivudine and zidovudine (Combivir® plus NVP) for those who cannot tolerate stavudine.

Children ages ≥ 3 to ≤ 12 will be enrolled in this study. Young children <5 years of age constitute the age group most likely to acquire malaria multiple times per year. Children <3 years will be excluded due to blood volume collection limitations. Children >12 years of age will be excluded as clinical pharmacokinetic studies suggest that the enzymatic activity of CYP or other metabolic enzymes is increased (although perhaps stable) in prepubescent children compared to either post-pubescent children or adults. Our primary objective is to evaluate the impact of nevirapine co-administration on the pharmacokinetics of artemether/lumefantrine. Thus the study will be restricted to

pre-pubescent children to minimize the number of potential confounders influencing our pharmacokinetic results (i.e. this will minimize the PK variability within the study). HIV-infected children managed with NVP will be enrolled in this study. To provide the best possible control group, children known to be HIV-positive but not yet on ARVs will also be enrolled. This will control for the effect of HIV infection on the PK study results. The children in the control group are not receiving ARVs because they do not meet national guidelines for needing such therapy.

2.0 STUDY OBJECTIVES

2.1 Primary Objectives

2.11 To determine if NVP-based ARV therapy versus no ARV therapy alters the AL PK exposure in children (ages ≥3 to ≤12 years) who are co-infected with malaria and HIV.

2.12 To evaluate the 28 and 42-day safety and toxicity following co-administration of 6 doses of AL with NVP. (Arm A only).

2.2 Secondary Objectives

2.21 To determine if HIV infection is associated with altered disposition of AL.

2.22 To explore the effects of malaria co-infection and artemisinin based treatment on HIV viral load in children who are and are not receiving ARV.

2.23 To explore the effects of malaria co-infection on selection of ARV drug resistance among children receiving NVP-based HAART and experiencing increase in viral loads.

2.24 To investigate the impact of AL on NVP pharmacokinetics.

2.25 To characterize the frequency and factors associated with early and late treatment failures.

3.0 STUDY DESIGN

3.1 Overview

P1079 is a prospective, PK study of 48 evaluable subjects, ages ≥3 to ≤12 years, who are co-infected with malaria and HIV. The study aims to investigate the drug-drug interaction between the antimalarial treatment artemether/lumefantrine (AL) and NVP-based ARV therapy for HIV, in co-infected subjects at sites in Africa with a high malaria burden. Two study arms will include children who are either on prescribed NVP-based ARV therapy (Arm A; n=24), or no ARV treatment (Arm B; n=24) at the time of presenting with non-severe malaria to sites that routinely treat pediatric malaria with AL. Note: See Section 4.27 for definition of severe malaria. Subjects must be receiving (a) NO ARV drugs for at least 4 weeks prior to study entry, OR (b) NVP-based combination ARV therapy for at least 4 weeks prior to study entry. Note: Children receiving NO ARV drugs are not yet on ARVs because they do not meet national guidelines for needing such therapy. Enrollment of subjects will be balanced by age at the time of malaria presentation, with the goal of achieving approximate balance of age distribution between arms in proportions of subjects above and below six years of age. (See Figure 1).

Figure 1: P1079 Enrollment Scheme

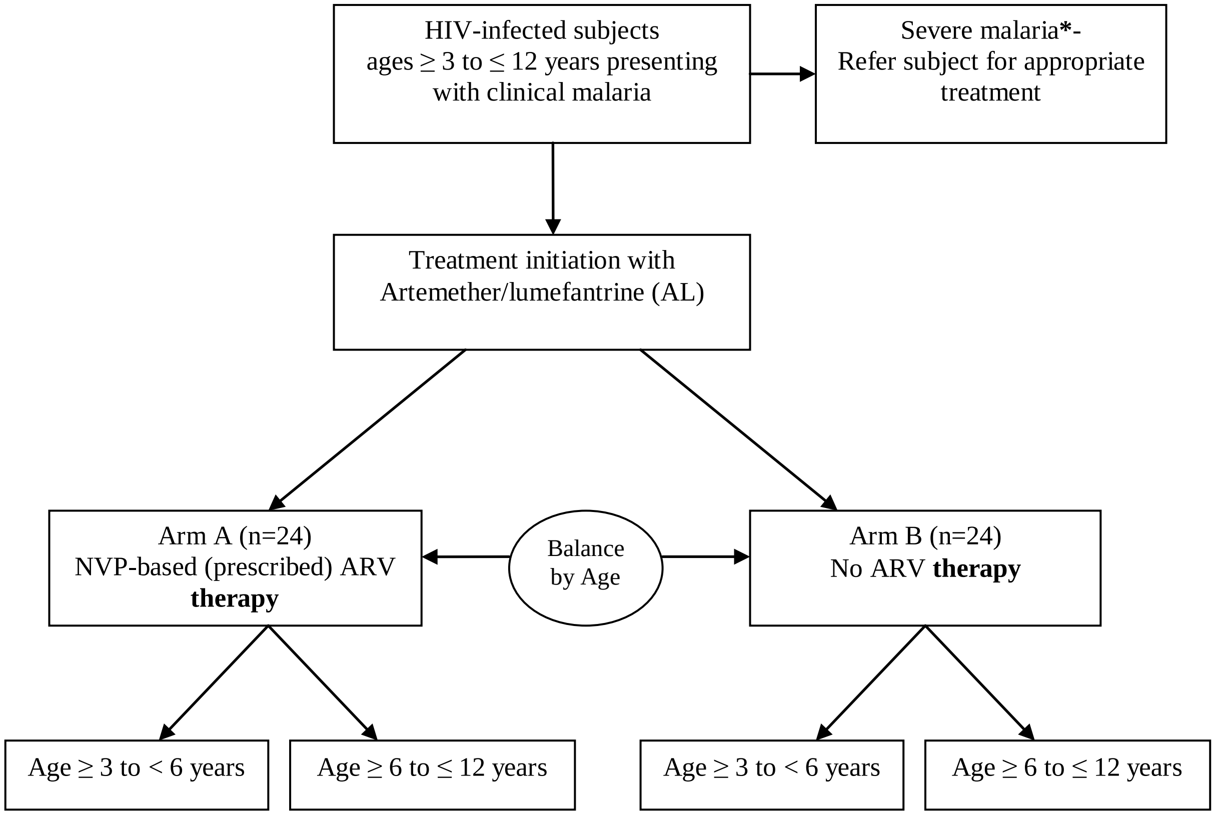

*** See Section 4.27 for definition of severe malaria.**

3.2 AL Dosing and Pharmacokinetics

Subjects who test positive for malaria will be given a total of 6 doses of AL, distributed over study Day 0 to study Day 3. Specifically, children will receive 2 weight-based doses of AL, approximately 8-12 hours apart on the day of study entry (study Day 0) and on study Day 2 of the study. On study Days 1 and 3, subjects will be given AL once daily. See Section 5.1 for AL dosing schedule. During AL treatment, subjects will also continue to receive the same NVP- based treatment prescribed by their physician (if any) for their HIV infection that they were receiving at time of malaria presentation. AL PK samples will be collected on study Day 3: Pre-dose, 1, 2, 4, and 8 hours; study Day 4: 24 hours; and study Day 8: 120 hours post-administration of the last AL dose.

3.3 NVP-Based ARV Therapy and Pharmacokinetics

Subjects will continue on NVP-based ARV therapy while receiving AL treatment. Two NVP PK samples will be collected; the first sample will be collected on Study Day 0 and the second sample on Study Day 3. The baseline (Study Day 0) pharmacokinetic sample of NVP will be collected at a random time (within 4 hours of initiation of study visit) prior to initiating AL treatment and following the previous NVP dose (approximately 12 hours following the evening dose of NVP). The second NVP sample, Study Day 3, will be collected just prior to the morning NVP dose and at the time of the last dose of AL.

All subjects will be followed for 42 days from study entry.

Refer to Appendix I for specific study requirements.

4.0 SELECTION AND ENROLLMENT OF SUBJECTS

4.1 Inclusion Criteria

4.11 Age ≥3 to ≤12 years at entry.

4.12 Documentation of HIV-1 infection defined as positive results from two samples collected at different time points. The same method may be used at both time points. All samples tested must be whole blood, serum or plasma.

Subjects ≤ 18 months of age

The first test may be any of the following:

- One HIV DNA PCR
- One HIV RNA (quantitative >5,000 copies/mL or qualitative)
- One HIV culture (prior to August 2009)
- One total HIV nucleic acid

If the first test(s) is positive, a second sample collected and tested using any of the tests listed above (except for qualitative RNA assays) in a laboratory participating in an appropriate external quality assurance program and NIH-approved.

Subjects > 18 months of age

The first test may be any of the following:

- Two rapid antibody tests from different manufacturers or based on different principles and epitopes
- One rapid antibody test AND one [enzyme immunoassay (EIA) OR Western blot (WB) OR immunofluorescence OR chemiluminescence]
- One EIA AND one [WB OR immunofluorescence OR chemiluminescence]
- One HIV DNA PCR
- One HIV RNA (quantitative >5,000 copies/mL or qualitative)
- One HIV culture (prior to August 2009)
- One total HIV nucleic acid

If the first test(s) is positive, a second sample collected and tested using any of the tests listed above (except for qualitative RNA assays) in a laboratory participating in an appropriate external quality assurance program and either CAP/CLIA approved (for US laboratories) or NIH-approved (for international laboratories).

4.13 Presentation with malaria as indicated by positive smear for malaria parasites along with clinical evidence of infection (fever or history of fever in the past 24 hours) with planned treatment with AL.

4.14 Receiving: (a) NO ARV drugs for at least 4 weeks prior to study entry with no intent to initate ARVs during the study duration, OR (b) NVP-based combination ARV therapy for at least 4 weeks prior to study entry, with the intent to continue same for duration of the study..

NOTE: Subjects who are managed with a NVP-based ARV therapy at the time of study enrollment will continue on NVP-based ARV therapy while receiving AL treatment. Children who have NOT met eligibility for ART according to national guidelines (based on <u>known available data at time of enrollment</u>) will be permitted to enroll in the study group of children receiving NO ARV drugs. For this study arm of NO ARV drugs, subjects must not be receiving any ARV drugs currently and they have must not have been on any ARV drugs for at least 4 weeks prior to entry.

4.15 If subject is already on antimalarial medication at time of study entry, no more than 3 doses of either <u>generic</u> AL or brand Coartem® (either standard tablet or the dispersible formulation) may be given prior to study entry. Subjects may have received only the first, second, and/or third dose as a different AL formulation from the study formulation of Coartem® Dispersible. However, the actual dose of artemether and lumefantrine that has been administered MUST be the same as that stipulated by the protocol.

4.16 Female subjects of reproductive potential (having reached menses, or not having reached menopause or not having undergone hysterectomy, bilateral oophorectomy, or tubal ligation) who engage in sexual activity that could lead to pregnancy must agree to avoid pregnancy during the entire 42 day trial and to consistently and appropriately use at least two of the following contraception methods: condoms, diaphragm or cervical cap with spermicide, IUD, hormonal-based contraception. A list of acceptable methods can be found in the FDA Birth Control Guide accessible at: http://www.fda.gov/womens .

Note: "Female subjects of reproductive potential" is defined as girls who have reached menarche or women who have not been post-menopausal for at least 24 consecutive months (e.g. who have had menses within the preceding 24 months), or have not undergone a sterilization procedure (hysterectomy, bilateral oophorectomy or salpingotomy). If the female subject is not of reproductive potential, she is eligible without requiring contraception.

4.17 Demonstrated ability and willingness to swallow study medications.

4.18 Parent or legal guardian able and willing to provide signed informed consent.

4.19 Ability and willingness to complete study procedures and follow-up at the same study site.

4.2 Exclusion Criteria

4.21 Subjects with ≥ Grade 3 hemoglobin abnormalities (toxicities will be graded by the DAIDS Table for Grading the Severity of Adult and Pediatric Adverse Events, Version 1.0, dated December 2004, Clarification August 2009, must be used and is available on the Regulatory Support Center (RSC) web site (http://rsc.tech-res.com/safetyandpharmacovigilance/).

4.22 Severe malnutrition will be defined as (i) body mass index (BMI) Z-score< -3SD for children ≥5 years old or (ii) Weight-for-Height <-3SD for children <5 years old. (See Appendix IV).

Note: Children will be evaluated for malnutrition at the time they present for study enrollment when screening evaluations are performed.

4.23 Receipt of a protease inhibitor or EFV within 4 weeks prior to study entry.

4.24 Subjects not on ART, but who qualify for ART, according to national guidelines (based on all data available at time of enrollment).

4.25 Use of AL for prior episode of malaria within 6 weeks of study entry.

4.26 Currently receiving an antimalarial drug other than AL.

4.27 Pregnancy or breastfeeding.

4.28 Signs or evidence of severe malaria. Severe malaria is defined as:

- Unarousable coma (if after convulsion, > 30 minutes)

OR ANY TWO OF THE FOLLOWING SYMPTOMS:

- Recent febrile convulsions (within 24 hours)
- Altered consciousness (confusion, delirium, psychosis, coma)
- Lethargy
- Unable to drink
- Unable to stand/sit due to weakness
- Severe anemia (Hb < 5.0 gm/dL)
- Respiratory distress (labored breathing at rest)
- Jaundice

4.29 Repeated vomiting that, in the opinion of the investigator, would interfere with oral administration and drug absorption.

4.210 Current treatment for malignancy.

4.211 Known allergy or intolerance to milk products.

4.212 In the case where a seemingly eligible participant who is small, has a known or planned blood draw, or will have blood drawn for any reason, such that the total volume blood being drawn over any 8 week period will exceed 9.5 mL/kg. (See Appendix II).

4.213 Any disallowed medications (see Section 4.3) used within 3 weeks of study entry.

Please contact the P1079 team [actg.teamp1079@fstrf.org] with questions.

4.3 Disallowed Medication Guidelines

The following medications are disallowed within 3 weeks prior to receiving study drug:

- Carbamazepine
- Clarithromycin
- Erythromycin (oral)
- Ketoconazole
- Phenobarbital
- Phenytoin
- Rifabutin
- Rifampin
- Halofantrine
- Any other medication known to significantly affect CYP450 metabolism.
- Grapefruit juice should be avoided during the study due to its potential effects on CYP3A4.

4.4 Enrollment Procedures

Prior to implementation of this protocol, and any subsequent full version amendments, each site must have the protocol document and the protocol consent form(s) approved, as appropriate, by their local Institutional Review Board (IRB)/Ethics Committee (EC) and any other applicable regulatory entity (RE). A Site Implementation Plan (SIP) is required from each site participating in the study. The plan must be submitted to the Protocol Team for review and approval before protocol registration can occur.

Upon receiving final approval, sites will submit all required protocol registration documents to the DAIDS Protocol Registration Office (DAIDS PRO) at the Regulatory Support Center (RSC). The DAIDS PRO will review the submitted protocol registration packet to ensure that all of the required documents have been received.

Site-specific informed consent forms (ICFs) *WILL* be reviewed and approved by the DAIDS PRO and sites will receive an Initial Registration Notification from the DAIDS PRO that indicates successful completion of the protocol registration process. A copy of the Initial Registration Notification should be retained in the site's regulatory files.

Upon receiving final IRB/EC and any other applicable RE approval(s) for an amendment, sites should implement the amendment immediately. Sites are required to submit an amendment registration packet to the DAIDS PRO at the RSC. The DAIDS PRO will review the submitted protocol registration packet to ensure that all the required documents have been received. Site-specific ICF(s) *WILL NOT* be reviewed and approved by the DAIDS PRO and sites will receive an Amendment Registration Notification when the DAIDS PRO receives a

complete registration packet. A copy of the Amendment Registration Notification should be retained in the site's regulatory files.

For additional information on the protocol registration process and specific documents required for initial and amendment registrations, refer to the current version of the DAIDS Protocol Registration Manual.

4.5 Co-enrollment Procedures

Co-enrollment is permitted where permitted by local/country regulations except for protocols that would violate the exclusion criteria. All co-enrollments in protocols require the approval from the P1079 Chair and the Chair of the co-enrollment study that the subject is interested in co-enrolling into.

5.0 STUDY TREATMENT

5.1 Drug Regimens, Administration and Duration

Drug Regimen: For the purposes of this protocol, only AL is considered to be a study drug. AL treatment regimen consists of 6 doses of study drug administered over 4 days. Subjects will be treated with study drug which is consistent with standard of care for malaria, and will receive a 6 dose course of AL as per weight-based dosing guidelines. The AL study drug to be administered is Coartem® Dispersible with the only exceptions specified in Section 4.15.

Drug Administration: Subjects may come to the clinic at variable times of the day and initiate treatment for their malaria. In order to assure that antimalarial treatment is carried out correctly and that PK assessments can occur during the day, rather than in the evening, the dosing schedule specified in Table 1 will be followed.

Dosage and Administration: Dispersible tablets for oral administration. The dispersible tablet(s) composing 1 dose should be completely dispersed in a small amount of water (approximately 10 mL per tablet). Stir gently and administer immediately to the subject. Rinse the glass with an additional small amount of water (approximately 10 mL) and give immediately to the subject. (See Appendix V).

For those subjects on their prescribed NVP-based ARV therapy, the doses of AL and NVP can be given together throughout the study and should be administered together on the day of the PK evaluations (around the last dose of AL).

Although subjects with acute malaria are frequently intolerant of food, AL should be followed by fluids (particularly drinks containing fats, such as milk) and if

possible, with a normal diet as soon as the subject can tolerate food. Ingestion with fat-containing food and drinks greatly improves absorption.

For this study, every dose of AL must be administered with whole milk as specified below. Although any risk of recrudescence is very small, subjects who remain averse to food during treatment should be closely monitored.

At the time of study entry, subjects' families will be provided with container(s) of whole milk or vouchers to purchase whole milk (such as: First Choice- full cream milk UHT process, which is from South Africa, with a fat content of 3.4g per 100 mL; Dairyfresh- Long life milk from Kenya, labeled whole milk with 3.5% fat; Crystal Valley- full cream milk, UHT process with 3.3g fat and is from Argentina; and/or Tuzo Long life whole milk with 3.5% fat from Kenya) to be administered with study drug in order to improve bioavailability.[11] Adherence of AL will be assured as each treatment dose time will be recorded. Most doses will be administered in the clinic and observed by a study nurse or at the subject's home and observed by the caregiver. Adherence for the NVP based regimen will also be monitored through subject logs.

If emesis occurs within one hour of administration, the dose should be repeated one time.

<table>
<tr><td colspan="7">Coartem® Dispersible (artemether/lumefantrine) dose administration

C= Clinic; H= Home</td></tr>
<tr><td></td><td colspan="2">STUDY DAY 0</td><td>STUDY DAY 1</td><td colspan="2">STUDY DAY 2</td><td>STUDY DAY 3</td></tr>
<tr><td></td><td>Dose 1</td><td>Dose 2</td><td>Dose 3</td><td>Dose 4</td><td>Dose 5</td><td>Dose 6</td></tr>
<tr><td colspan="7">Enrollment at Time Malaria Treatment is Initiated</td></tr>
<tr><td></td><td>1100–1400 Hours (11AM -2PM) (C)</td><td>2100-2400 Hours (9PM -12AM) (H)</td><td>1300-1500 Hours (1PM -3PM) (C)</td><td>0800-1000 Hours (8AM -10AM) (C)</td><td>2000-2200 Hours (8PM-10PM) (H)</td><td>0800-1000 Hours (8AM-10AM) (C)</td></tr>
<tr><td></td><td>1400-1700 Hours (2PM -5PM) (C)</td><td>2200-0100 Hours (10PM-1AM) (H)</td><td>1300-1500 Hours (1PM -3PM) (C</td><td>0800-1000 Hours (8AM -10AM) (C)</td><td>2000-2200 Hours (8PM-10PM) (H)</td><td>0800-1000 Hours (8AM-10AM) (C)</td></tr>
<tr><td colspan="7">Enrollment after Malaria Treatment is Initiated**</td></tr>
<tr><td>After Dose 1</td><td></td><td>Give 2nd dose 8-12 hrs after first dose (C or H)</td><td>Give 3rd dose 12-16 hours after 2nd dose (C or H)*</td><td>0800-1000 Hours (8AM -10AM) (C)</td><td>2000-2200 Hours (8PM-10PM) (H)</td><td>0800-1000 Hours (8AM-10AM) (C)</td></tr>
<tr><td>After Dose 2</td><td></td><td></td><td>Give 3rd dose 12-16 hours after 2nd dose (C or H)*</td><td>0800-1000 Hours (8AM -10AM) (C)</td><td>2000-2200 Hours (8PM-10PM) (H)</td><td>0800-1000 Hours (8AM-10AM) (C)</td></tr>
<tr><td>After Dose 3</td><td></td><td></td><td>3rd dose must have been taken in the afternoon (1</td><td>0800-1000 Hours (8AM -10AM) (C)</td><td>2000-2200 Hours (8PM-10PM) (H)</td><td>0800-1000 Hours (8AM-10AM) (C)</td></tr>
</table>

			PM or later) to allow 4th dose to be administered between 0800-1000 Hours (8AM -10AM)			
After 4th or 5th dose: Not eligible for enrollment						
*The timing of this dose ideally should be 1 PM or later to allow 4th dose to be administered between 0800-1000 Hours (8 AM-10 AM)<br>** See Section 4.15.<br><br>NOTE: In the event of vomiting within 1 hour of administration a repeat dose should be taken.						

Table 1: Coartem® Dispersible (artemether/lumefantrine) dose administration schedule

Subjects who come on study after their first dose of medication should complete their 6 dose regimen as per Table 1.

Table 2: Coartem® Dispersible (artemether/lumefantrine) dosing

Weight	Coartem® Dispersible 20mg /120 mg tabs
<15 kg	1
≥15 to <25 kg	2
≥25 to <35 kg	3
≥35 kg	4

5.2 Drug Formulation

The dispersible formulations of AL contain 20 mg artemether, 120 mg of lumefantrine (Coartem® Dispersible).

Flat, yellow tablet with bevelled edges; imprinted: "CD" on one side and "NVR" on other side.

List of excipients: Microcrystalline cellulose, crospovidone, cherry dry flavour, croscarmellose sodium, saccharin sodium (8 mg / dispersible tablet), magnesium stearate, hypromellose, silica colloidal anhydrous, polysorbate 80.

Protect from heat and moisture; do not store above 30°C.

Subjects who are receiving NVP may receive fixed dose combinations of NVP, stavudine, and lamivudine, and may be receiving NVP tablets or NVP suspension.

5.3 Drug Supply, Distribution and Pharmacy

ARVs, including NVP, will be supplied to subjects as part of their background antiretroviral therapy and are not provided as part of the study.

Necessary funding for purchase of the Coartem® Dispersible formulation of AL will be provided by the study to participating study sites. Each study site must determine how to obtain Coartem® Dispersible. Coartem® Dispersible will be administered to ensure consistency in terms of drug delivery. However, some subjects may have treatment initiated at a rural clinic where a generic formulation of AL is available. Therefore subjects may have received only the first, second, and/or third dose as a different formulation as long as the actual AL dose administered is the same as that stipulated by the protocol (Section 5.1).

The IMPAACT pharmacist is required to maintain complete records of the AL dispensed to participants in this study as outlined in the manual *Pharmacy Guidelines and Instructions for DAIDS Clinical Trials Networks* in the section Study Product Management Responsibilities.

AL will not be supplied through the NIAID Clinical Research Products Management Center. AL is not to be included on the DAIDS Study Product Destruction Forms.

6.0 SUBJECT MANAGEMENT

6.1 Toxicity Management

The DAIDS Table for Grading the Severity of Adult and Pediatric Adverse Events, Version 1.0, dated December 2004, Clarification August 2009, must be used and is available on the RSC web site (http://rsc.tech-res.com/safetyandpharmacovigilance/).

Management of adverse experiences will be according to the best clinical practice and the judgment of the site investigator. Alternate explanations for clinical and laboratory abnormalities must be sought. Laboratory normals will be the institutional values. However, if a site does not have an age-specific normal range/value for a particular laboratory evaluation, the site should use the latest edition of the Harriet Lane Handbook for normal ranges/values and document this for monitoring purposes. Abnormal clinical and laboratory findings that develop during study participation should be followed until resolution to < Grade 2.

The toxicity management guidelines are for events for which a relationship to study drugs cannot be excluded. Clinical or laboratory adverse events (AEs) that are definitely unrelated to study drug(s) may not result in study drug interruption.

For the purpose of this study, study drug should not be held or modified for pre-existing Grade 3 or 4 laboratory toxicity. For this study, the study drug, AL, prescribed by the subject’s primary clinician, is used to treat acute malaria, and is under the direction of this study’s investigators. Once a child is enrolled in P1079, the site physicians (in conjunction with the subject’s primary clinician) will be responsible for AL treatment and toxicity management.

Subjects with Grade 3 or 4 hemoglobin at entry are allowed to participate in all study procedures (e.g. evaluation of the 28 and 42-day safety and toxicity) EXCEPT for the AL PK sampling or single samples for NVP measurements (to mimimize the volume of blood drawn in these children). They will, however, participate in follow-up safety visits at study days 14, 28 and 42, per Appendix I. Subjects showing evidence of Grade 3 or 4 neutropenia or thrombocytopenia or thrombocytosis at either screening or entry will be allowed to complete all study evaluations.

Relationships between drug exposure and study Day 28 and 42 outcomes will be evaluated. Subjects will also be assessed for any Grade 3 or 4 toxicities including neutropenia, other hematological abnormalities, abdominal pain, jaundice, nausea, vomiting, vertigo, dizziness, seizures, or change in mental status. These toxicities do not mandate discontinuation of AL. AL should be discontinued for any Grade 4 toxicity that, in the site investigator's/clinician's opinion, will be harmful to continue the subject on study drug. The P1079 team [actg.teamp1079@fstrf.org] requests to be notified within 24 hours of event.

ARV Therapy

Subjects on study will have management of their ARV therapy as it relates to Grades 2, 3, and/or 4 laboratory assessments as per their primary care provider. Data from screening and entry as well as other laboratory assessments will be made available to the primary care provider as they become available. All attempts will be made to notify the primary care provider of all Grade 4 toxicity evaluations within 72 hours.

6.2 Study Management Plan

HIV-infected children presenting to clinics with symptoms suggestive of malaria (fever or history of fever in the past 24 hours) will undergo standard procedures for the diagnosis of malaria. Those who are smear positive for malaria parasites will be treated according to local antimalarial treatment protocol with AL. Subjects who meet criteria for severe malaria (see Section 4.27) will not be enrolled and will be directed to clinic physicians for appropriate therapy. At the time of presentation with malaria, study physicians will approach and explain the study to parents or guardians of children presenting to the clinic. Children who meet entry criteria will be enrolled per the Data Management Center's Subject Enrollment System, and treated according to the study design after obtaining consent from the parent or guardian.

Children will continue to receive comprehensive care for HIV through their primary health care provider. They will be evaluated monthly according to National/WHO guidelines where antiretroviral therapy will be initiated accordingly. If results are gained during the course of the study that are relevant to HIV care for a participating child, this information will immediately be provided to the child's primary health care provider. If it is deemed in the best interest of the child to initiate antiretroviral treatment during the course of the study, children will discontinue their participation in P1079. Children will not be followed for their HIV care through P1079. These subjects who discontinue their participation prior to Day 28 will be replaced.

This study is to evaluate the pharmacokinetics of AL after 3 days of treatment. However, children will be followed for 42 days. In the event a girl becomes

pregnant after enrollment, she must complete treatment for clinical malaria. She will immediately be referred for antepartum clinical care. If feasible, she will be permitted to continue on study and complete the PK sampling. Any data related to pregnancy will be captured on the case report form. The P1079 protocol team [actg.teamp1079@fstrf.org] must be notified within 72 hours.

6.21 Initial Screening and Diagnostics

6.211 Thick Blood Smears

Subjects will be referred to the outpatient laboratory for screening thick blood smear, obtained by fingerprick, using standard Giemsa staining. Thick blood slides will be read and counted by the laboratory technicians at the time of presentation. The parasite density of positive screening thick blood smears will be estimated by counting the number of asexual parasites per 200 leukocytes, assuming a leukocyte count of 8,000/µl. Smears will be considered negative when examination of 100 high-power fields does not reveal parasites. The thick smear must be seen by the designated reader at each site prior to the Day 3 PK study day evaluations indicated in Appendix I.

6.212 Thin Blood Smears

Thin blood smears will be evaluated for parasite species. All subjects with a positive smear parasite density of 2000-200,000/µL will be referred to the PK study physicians to determine further eligibility for study enrollment.

6.213 Diagnostics

Both thick and thin blood smears will be transferred to the primary clinical site and read by a designated reader (to be identified at each site) for verification.

Subjects who are diagnosed with malaria will be referred to study physicians for a screening interview. If the subject appears to be an appropriate candidate for study enrollment, the informed consent process will be initiated (study Day 0). Consent can also be obtained for the PK study after initiating antimalarial drugs but the total doses of drug administration will not exceed 3 doses. If the subject does not meet entry criteria or consent is not obtained, the child will be referred for standard clinical care.

6.214 DNA Fingerprinting of Parasites

For all repeat episodes of malaria, molecular genotyping methods will be used to distinguish recrudescence (recurrent malaria due to parasite strains present at the time of the previous episode of malaria) from new infections (recurrent malaria due to newly infecting parasite strains) by methods which have been well validated in our laboratory at UCSF [36]. DNA fingerprinting is performed using capillary electrophoresis with optimized PCR reaction and cycling conditions. Samples are run on an Applied Biosystems 3730xl DNA Analyzer and DNA sizes are compared with GeneMapper 4.0 software (Applied Biosystems). Molecular studies will be performed only for research purposes and will have no impact on the immediate clinical management of study patients. Molecular genotyping will be performed on a single dried blood spot in the laboratory of Dr. Sunil Parikh at UCSF.

6.22 Adherence Assessment

The date, time and formulation of all doses of AL and NVP (both at home and at clinic) will be recorded at the study sites on the protocol CRF. Whether or not a subject missed a dose and the date and time of the missed dose should be recorded on the CRF. If a subject misses any AL or NVP dose within the 48 hours preceding the intensive PK sampling period, he/she will not be allowed to participate in the PK sampling procedures as missed doses will affect the PK parameter estimates (see section 9.2).

6.3 Criteria for Early/Late Treatment Failure

Subjects meeting criteria for early or late treatment failure as per the following will be treated as per standard protocol for malaria management (e.g. quinine treatment for early treatment failure, clinical failure). Children who experience *early treatment failure* requiring quinine treatment may be instructed to discontinue AL therapy and will not undergo PK sampling. Children experiencing *clinical failure* will complete all study related procedures.

6.31 Early Treatment Failure: Study Days 0, 1, 2, and 3

- Development of danger signs or severe malaria on study Days 0-3 in the presence of parasitemia
- Parasitemia on study Day 2 higher than on Day 0, irrespective of temperature
- Parasitemia on study Day 3 with temperature > 38.0º C (tympanic)
- Parasitemia on study Day 3 > 25% of count on Day 0

6.32 Clinical Failure: Study Days 4 to 28

- Presence of parasitemia on study Days 4-28 with temperature > 38.0º C (tympanic) or history of fever in past 24 hours
- Development of danger signs or severe malaria study Days 4 to 28 in the presence of parasitemia

6.33 Adequate Clinical and Parasitological Response

- Absence of parasitemia on study Day 28, with no prior requirement for additional treatment other than the standard 6 dose regimen received at time of presentation with malaria.
- For subjects who experience treatment failure before study Day 3 (see Section 6.31), PK sampling will be discontinued.

6.4 Criteria for Study Drug (AL) Discontinuation

- Treatment with any disallowed medication listed in Section 4.3
- Drug toxicity that requires permanent study drug discontinuation as defined in Section 6.1
- Nonadherence (defined in Section 6.22)

Subjects who discontinue study treatment will continue to be followed at scheduled study visits.

6.5 Criteria for Study Discontinuation

- The subject or legal guardian refuses further treatment and/or follow-up evaluations.
- The investigator determines that further participation would be detrimental to the subject's health or well-being.
- If the subject requires initiation of antiretroviral treatment during the course of the study (see Section 6.2).
- The subject fails to comply with the study requirements so as to cause harm to him/herself or seriously interfere with the validity of the study results.

7.0 ADVERSE EVENT REPORTING

7.1 Adverse Event Reporting to DAIDS

Requirements, definitions and methods for expedited reporting of Adverse Events (AEs) are outlined in Version 2.0 of the DAIDS EAE Manual, which is available on the RSC website at http://rsc.tech-res.com/safetyandpharmacovigilance/.

The DAIDS Adverse Experience Reporting System (DAERS), an internet-based reporting system, must be used for expedited AE reporting to DAIDS. In the event of system outages or technical difficulties, expedited AEs may be submitted via the DAIDS EAE Form. For questions about DAERS, please contact DAIDS-ES at DAIDS-ESSupport@niaid.nih.gov. Site queries may also be sent from within the DAERS application itself.

Where DAERS has not been implemented, sites will submit expedited AEs by documenting the information on the current DAIDS EAE Form. This form is available on the RSC website: http://rsc.tech-res.com/safetyandpharmacovigilance/. For questions about EAE reporting, please contact the RSC (DAIDSRSCSafetyOffice@tech-res.com).

7.2 Reporting Requirements for this Study

- The SAE EAE Reporting Category, as defined in Version 2.0 of the DAIDS EAE Manual, will be used for this study.
- The study agents for which expedited reporting are required are: artemether/lumefantrine.

7.3 Grading Severity of Events

DAIDS Table for Grading the Severity of Adult and Pediatric Adverse Events, Version 1.0, dated December 2004, Clarification August 2009, must be used and is available on the RSC web site (http://rsc.tech-res.com/safetyandpharmacovigilance/).

7.4 Expedited AE Reporting Period

- The expedited AE reporting period for this study is the entire study duration for an individual subject (from study enrollment until study completion or discontinuation of the subject from study participation for any reason).
- After the protocol-defined AE reporting period, unless otherwise noted, only SUSARs as defined in Version 2.0 of the EAE Manual will be reported to

DAIDS if the study staff become aware of the events on a passive basis (from publicly available information).

8.0 STATISTICAL CONSIDERATIONS

8.1 General Design Issues

This is a prospective PK study to investigate the drug-drug interaction between NVP-based ARV and the antimalarial drug AL in HIV-malaria co-infected subjects. This study will also examine the effects of AL and malaria co-infection on HIV viral load in subjects who are and are not receiving ARV. The study will enroll subjects aged ≥3 to ≤12 years, who will be assigned to one of the two study arms depending on whether they are receiving ARV therapy or not. Enrollment will be constrained so that at least 8 patients are present in each age stratum on each arm. Subjects will be replaced if pharmacokinetic results are deemed un-evaluable by the study team (for example, mislabeled specimens, multiple samples that have insufficient quantity, contaminated and/or mistimed samples, etc.), or if a subject on Arm B (i.e. not receiving ARVs) must initiate ARV treatment prior to Day 28.

8.2 Outcome Measures

Primary outcome measures are standard noncompartmental PK estimates including:

- the area under the plasma concentration versus time curve (AUC)
- minimum concentration (C_{min}), and peak concentration (C_{max}), computed for each individual and then summarized for strata
- number and percent of subjects with ≥Grade 3 adverse events will be recorded.

Secondary outcome measures include:

- HIV-1 viral load
- NVP resistance

8.3 Randomization and Stratification

This is an observational study. There is no randomization. The intended design has 12 subjects aged <6 years and 12 subjects aged ≥6 years on each arm. However, the enrollment plan allows some flexibility in the actual fractions of each age group, provided that, no less than 8 subjects and no more than 16 subjects are enrolled in each age group. Standard DMC enrollment stratification monitoring facilities will be used to verify prospectively that each attempted enrollment satisfies these conditions.

The team will assess accrual on a quarterly basis. If any age group in any arm has not accrued half of its subjects within 12 months after opening of the protocol, the team will identify the reasons for lack of accrual and take corrective actions as needed.

8.4 Sample Size and Accrual

Twenty-four subjects will be enrolled in each of the two arms for a total of 48 subjects. Assuming equal variances in both arms, shown below are the required sample sizes in each arm to detect the specified difference in mean AUC with 80% power and a two-sided Type I error of 5% for a range of CVs. Based on this table, we estimate 23 subjects will be needed in each arm to detect a difference in mean AUC of 30% assuming a CV of 35%. To assure even numbers of subjects per arm, a total sample size of 48 subjects (24 per arm) is selected.

Table 3: Sample size selection

CV	Delta: 25%	30%	35%
28%	21	15	12
35%	32	23	17
42%	46	32	24
50%	64	45	34

Since the second primary objective is to evaluate the 28 and 42-day safety and toxicity following co-administration of 6 doses of AL with NVP, only the 24 subjects in Arm A will be evaluated for the number of ≥Grade 3 adverse events. For a sample size of 24, Figure 2 below demonstrates the expected width of the 95% confidence interval for the probability of a safety event as a function of the true safety event rate. For example, the expected width of the 95% confidence interval for the probability of a safety event is 0.16 if the true safety event rate is 1%, 0.21 if the true rate is 5%, and 0.34 if the true rate is 20%.

Figure 2: Expected 95% Confidence Intervals for the Probability of a Safety Event (N=24)

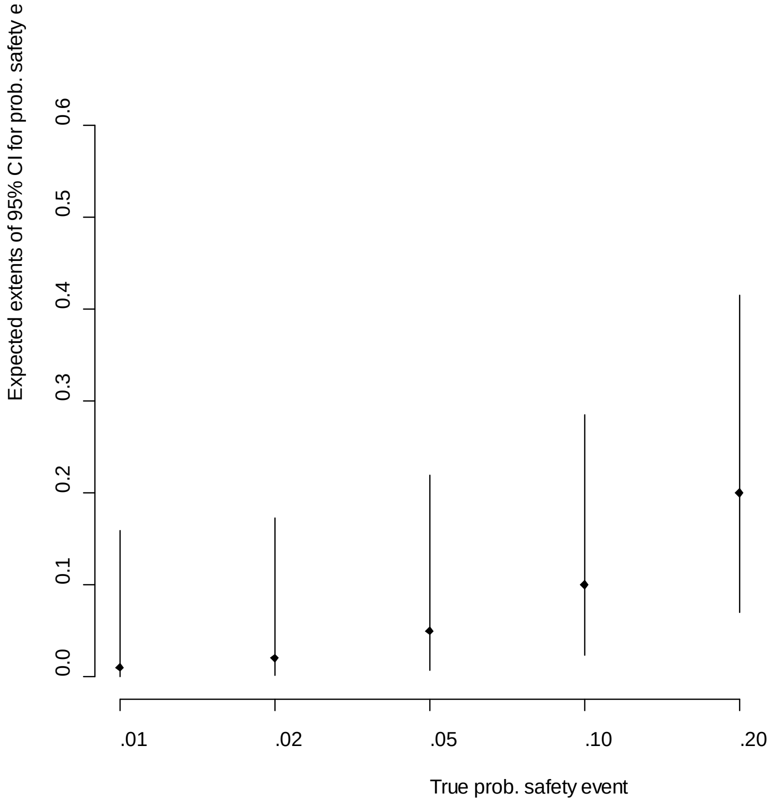

8.5 <u>Monitoring</u>

In order to make the team aware of the study's progress in an ongoing manner, a monitoring plan will be developed to identify which data items will be reviewed by the team and what the timing of such routine reviews will be. Monthly conference calls will be held by the study team to assess accrual of subjects and to review follow-up statistics reported to the team by the data management group. Rate of enrollment will be closely monitored and in the case of slower than expected accrual, the team will try to identify the reasons for lack of enrollment and, with input provided from the participating sites, provide solutions to speed up enrollment. Protocol violations and adverse events, if any, will be enumerated in these reports. Sections 6 and 7 above detail the formalities of adverse event reporting. Monthly team calls will be conducted after the first subject has enrolled, with frequency to be adjusted as needed as the study progresses. In the event of a death, the site must submit a CRF within 48 hours, and must notify the RSC within 72 hours. In addition, the protocol team and DAIDS Medical Officer

(MO) will review the death event and discuss it with the site principal investigator.

8.6 Analyses

Non-compartmental PK analysis is carried out using WinNonlin® to estimate the AUC and other parameters of the concentration time profile.

- Analysis for primary objective 1 consists of comparing PK parameters for AL between subjects who are receiving NVP-based ARV therapy (Arm A) and those who are not on ARV treatment (Arm B). Appropriate two-sample tests such as t-test or ANOVA will be used. If PK parameters are found to have skewed distributions and data transformations do not induce symmetry, rank-based tests will be used. Also, given the observational character of the study, it is possible that subjects on arms A and B differ with respect to features that could impact the pharmacokinetics of AL. We will perform a thorough exploratory data analysis of possibly relevant covariates and will include descriptive information in the analysis report. We will check for sensitivity of major PK inferences to adjustment for important covariates by including these in nonlinear models, and will compute propensity scores for assignment to arms A and B and employ this score as a covariate permitting concise representation of heterogeneities in the basic PK models. PK models that include the propensity score will be regarded as more reliable tools for comparison of subjects on arms A and B in the observational context.

- Analysis for primary objective 2 consists of evaluating the safety of the co-administration of 6 doses of AL and NVP by summarizing the number and percent of subjects with documented Grade 3 or higher adverse events.

- Analysis for secondary objective 1 consists of computation of confidence intervals for AL exposure among subjects who are co-infected with malaria and HIV and are not receiving any ARV treatment (Arm B) and comparing these to historical values for HIV-negative subjects who have malaria.

- Analysis for secondary objective 2 consists of computation of confidence intervals for mean log10 HIV RNA copy number for children at different treatment stages: at baseline, when malarial illness may be associated with elevated HIV RNA load; subsequent to AL treatment, when malarial illness is eliminated; and during AL treatment. Both cohort-averaged and within-individual changes will be assessed in these exploratory analyses.

- Analysis for secondary objective 3 consists of comparison of resistance mutation frequencies among individuals with increasing viral load who are receiving NVP-containing HAART with population mutation frequency estimates in comparable pediatric populations who are not infected with malaria.

- Analysis for secondary objective 4 consists of computation of confidence intervals for the mean within-person difference between NVP PK trough levels taken at baseline in the absence of AL and NVP trough levels collected at the time of the last AL dose administration (see Section 9.22, Pharmacokinetic Analysis).

- Analysis for secondary objective 5 consists of summarizing the number and percent of subjects with early and late antimalarial treatment failure and comparing HIV-related, malaria-related, and demographic factors between early- and late-failing subjects. Malaria-related factors include level of parasitemia and hematocrit and HIV-related factors include viral load, absolute lymphocyte count, and antiretroviral treatment regimen.

9.0 CLINICAL PHARMACOLOGY PLAN

9.1 Pharmacology Objectives

This is a pharmacology study and the study objectives are as outlined in Section 2.0.

9.2 Study Design, Modeling and Data Analysis

Study procedures will be followed as outlined in Sections 3.0 to 5.0.
Subjects will receive 6 doses of AL as per the dosing guidelines specified in Secion 5.1. Subjects who are receiving a NVP based ARV regimen, will have been on the regimen for at least 4 weeks prior to study entry and subjects who are on no ARVs, must not have been on ARVs for at least 4 weeks prior to study entry. To assure that AL absorption is optimized and consistent; subjects will receive whole milk, pasteurized, shelf-stable to be taken with each AL dose administration (product specified in section 5.1). Dosing will be scheduled so that the 6th dose is administered in the morning to permit intensive PK sampling during the day rather than during the evening. The PK sampling procedures will take place in either a clinic or hospital setting that is equipped to handle proper sample collection and processing. All pharmacokinetic samples will be registered in the Lab Data Management System (LDMS).

9.21 Pharmacokinetic Sampling:

9.211 AL Sampling:

Pharmacokinetics of AL will be collected around the last treatment dose for all subjects enrolled in the study to compare the PK of AL in subjects who are receiving a NVP-based regimen for their HIV to subjects who are not receiving any ARVs for their HIV (Primary Objective 2.11). Plasma samples will be collected for AL PK on study Day 3: Pre-dose, 1, 2, 4, and 8 hours; study Day 4: 24 hours; and study Day 8: 120 hours post-administration of the last AL dose.

9.212 NVP Sampling:

Two NVP PK samples will be collected. Samples for NVP will be collected at baseline (at the time they are diagnosed with malaria and prior to initiating AL treatment) and at the time of the last dose of AL to explore the impact of AL on NVP exposure.

A baseline level of NVP will be collected at the time subjects come to clinic and will occur at a random time following the previous NVP dose. A second NVP sample will be collected at the time of the last dose of AL (approximately 12 hours following the evening dose of NVP). (Secondary Objective 2.24). This second NVP sample is collected just prior to the morning NVP dose and at the time subjects come to the clinic to undergo their serial PK sampling.

9.22 Pharmacokinetic Analysis:

From the intensive PK sampling for AL, PK parameters will be estimated. Non- compartmental analysis will be performed for individual PK estimates via the linear up-log down trapezoidal rule in conjunction with an oral input model using WinNonlin 5.0.1 (Pharsight Corporation, Mountain View, USA). PK parameters, including elimination rate constant (λz) and half life ($t_{1/2}$) will be estimated with $t_{1/2}$ calculated as ln2/ λz. The AUC will be estimated as AUC_{last} (AUC to the end of the sampling period) and $AUC_{0\text{-}inf}$ for artemether (AR), dihydroartemisinin (DHA) and lumefantrine.

For NVP, samples collected at baseline at random times will be corrected based on prior population PK estimates so that the value represents a trough or 12 hour NVP concentration. This way the baseline NVP

measurement can be compared to the trough (12 hour) measurement collected at the time of the last AL dose administration.

All samples for AR, DHA, lumefantrine and NVP analysis will be shipped to and analyzed at the IMPAACT Pharmacology Laboratory at the University of California, San Francisco. Validated analytical procedures will be used to quantitate AR, DHA, lumefantrine, and NVP. All methods are approved by the DAIDS sponsored Pharmacology Quality Assurance Program (PQA). Detailed collection, processing, and shipping information can be found in the Laboratory Processing Chart (LPC).

9.3 Virologic Sampling

Blood samples will be collected and stored as dried blood spots for viral load measurements at entry (study Day 0), and study Days 8, 14, and 42, and for drug resistance testing at entry and study Day 42.

10.0 HUMAN SUBJECTS

10.1 Institutional Review Board and Informed Consent

This protocol, the informed consent document (Appendix VI), and any subsequent modifications must be reviewed and approved by the IRB or Ethics Committee (EC) responsible for oversight of the study. Written informed consent must be obtained from the subject’s parents or legal guardians. The subject's assent must also be obtained if he or she is able to understand the nature, significance, and risks of the study. The informed consent will describe the purpose of the study, the procedures to be followed, and the risks and benefits of participation. A copy of the consent form will be given to the subject’s parent or legal guardian.

Each site which receives US HHS funding and follows the United States Code of Federal Regulations Title 45-Public Welfare, Part 46-Protection of Human Subjects (also known as the Common Rule) should have on record at the site a plan that detects and addresses any change in guardianship occurring in pediatric subjects and determines when a study subject must have a consent process which involves a legally authorized representative (LAR) other than a family member with guardianship. The plan will include how the site determines when a LAR is initially or no longer needed and how frequently the LAR re-signs the consent. The plan should follow all IRB/EC, local, state, national and/or host country guidelines. Confirmation of such a plan at a site should be submitted with protocol registration materials.

10.2 Subject Confidentiality

All laboratory specimens, evaluation forms, reports, and other records will be identified only by a coded number to maintain subject confidentiality within the research record. All records will be kept in a secured area. All computer entry and networking programs will be done with coded numbers only. Clinical information will not be released without written permission of the subject, except as necessary for monitoring by the Office for Human Research Protections (OHRP), the NIAID, the local IRB or EC.

10.3 Study Discontinuation

The study may be discontinued at any time by the NIAID, the IRB or EC, OHRP, IMPAACT Network, or other country-specific governmental agencies as part of their duties to ensure that research subjects are protected.

11.0 PUBLICATION OF RESEARCH FINDINGS

Publication of the results of this trial will be governed by IMPAACT policies.

12.0 BIOHAZARD CONTAINMENT

As the transmission of HIV and other bloodborne pathogens can occur through contact with contaminated needles, blood, and blood products, appropriate blood and secretion precautions will be employed by all personnel in the drawing of blood and shipping and handling of all specimens for this study, as currently recommended by the Centers for Disease Control and Prevention.

All infectious specimens will be sent using packaging that meets requirements specified by the International Air Transport Association Dangerous Goods Regulations for UN3373, Biological Substance, Category B, and Packing Instruction 650. Refer to individual carrier guidelines (e.g., Federal Express or Airborne) as well as specific requirements of the host country for specific instructions required for ground transportation within that country.

13.0 REFERENCES

(1) Pickering LK. AAP 2006 Report of the Committee on Infectious Diseases. 27th ed. 2006.

(2) World Health Organization. WHO Briefing on Malaria Treatment guidelines and artemisinins monotherapies. 2006. Geneva.

(3) Gasasira AF, Kamya MR, Achan J et al. High risk of neutropenia in HIV-infected children following treatment with artesunate plus amodiaquine for uncomplicated malaria in Uganda. Clin Infect Dis 2008; 46(7):985-991.

(4) Dorsey G, Gasasira AF, Machekano R, Kamya MR, Staedke SG, Hubbard A. The impact of age, temperature, and parasite density on treatment outcomes from antimalarial clinical trials in Kampala, Uganda. Am J Trop Med Hyg 2004; 71(5):531-536.

(5) Bukirwa H, Yeka A, Kamya MR et al. Artemisinin combination therapies for treatment of uncomplicated malaria in Uganda. PLoS Clin Trials 2006; 1(1):e7.

(6) Martensson A, Stromberg J, Sisowath C et al. Efficacy of artesunate plus amodiaquine versus that of artemether-lumefantrine for the treatment of uncomplicated childhood Plasmodium falciparum malaria in Zanzibar, Tanzania. Clin Infect Dis 2005; 41(8):1079-1086.

(7) German PI, Aweeka FT. Clinical pharmacology of artemisinin-based combination therapies. Clin Pharmacokinet 2008; 47(2):91-102.

(8) Ilett KF, Ethell BT, Maggs JL et al. Glucuronidation of dihydroartemisinin in vivo and by human liver microsomes and expressed UDP-glucuronosyltransferases. Drug Metab Dispos 2002; 30(9):1005-1012.

(9) Cumming JN, Ploypradith P, Posner GH. Antimalarial activity of artemisinin (qinghaosu) and related trioxanes: mechanism(s) of action. Adv Pharmacol 1997; 37:253-297.

(10) Lefevre G, Carpenter P, Souppart C, Schmidli H, McClean M, Stypinski D. Pharmacokinetics and electrocardiographic pharmacodynamics of artemether-lumefantrine (Riamet) with concomitant administration of ketoconazole in healthy subjects. Br J Clin Pharmacol 2002; 54(5):485-492.

(11) Ashley EA, Stepniewska K, Lindegardh N et al. How much fat is necessary to optimize lumefantrine oral bioavailability? Trop Med Int Health 2007; 12(2):195-200.

(12) Floren LC, Wiznia A, Hayashi S et al. Nelfinavir pharmacokinetics in stable human immunodeficiency virus-positive children: Pediatric AIDS Clinical Trials Group Protocol 377. Pediatrics 2003; 112(3 Pt 1):e220-e227.

(13) Cooney GF, Habucky K, Hoppu K. Cyclosporin pharmacokinetics in paediatric transplant recipients. Clin Pharmacokinet 1997; 32(6):481-495.

(14) Bartelink IH, Rademaker CM, Schobben AF, van den Anker JN. Guidelines on paediatric dosing on the basis of developmental physiology and pharmacokinetic considerations. Clin Pharmacokinet 2006; 45(11):1077-1097.

(15) Hunt A, Joel S, Dick G, Goldman A. Population pharmacokinetics of oral morphine and its glucuronides in children receiving morphine as immediate-release liquid or sustained-release tablets for cancer pain. J Pediatr 1999; 135(1):47-55.

(16) Mwesigwa J, Parikh S, McGee B et al. Pharmacokinetics of artemether-lumefantrine and artesunate-amodiaquine in children in Kampala, Uganda. Antimicrob Agents Chemother 2010; 54(1):52-59.

(17) Checchi F, Piola P, Fogg C et al. Supervised versus unsupervised antimalarial treatment with six-dose artemether-lumefantrine: pharmacokinetic and dosage-related findings from a clinical trial in Uganda. Malar J 2006; 5:59.

(18) Price RN, Uhlemann AC, van VM et al. Molecular and pharmacological determinants of the therapeutic response to artemether-lumefantrine in multidrug-resistant Plasmodium falciparum malaria. Clin Infect Dis 2006; 42(11):1570-1577.

(19) Ribeiro IR, Olliaro P. Safety of artemisinin and its derivatives. A review of published and unpublished clinical trials. Med Trop (Mars) 1998; 58(3 Suppl):50-53.

(20) van VM, Angus BJ, Price RN et al. A case-control auditory evaluation of patients treated with artemisinin derivatives for multidrug-resistant Plasmodium falciparum malaria. Am J Trop Med Hyg 2000; 62(1):65-69.

(21) Bakshi R, Hermeling-Fritz I, Gathmann I, Alteri E. An integrated assessment of the clinical safety of artemether-lumefantrine: a new oral fixed-dose combination antimalarial drug. Trans R Soc Trop Med Hyg 2000; 94(4):419-424.

(22) German P, Greenhouse B, Coates C et al. Hepatotoxicity due to a drug interaction between amodiaquine plus artesunate and efavirenz. Clin Infect Dis 2007; 44(6):889-891.

(23) World Health Organization, UNICEF, UNAIDS. HIV and infant feeding counselling: a training course. Trainers' Guide. 2000.

(24) World Health Organization. The roll back malaria partnership. 2006.

(25) Birku Y, Mekonnen E, Bjorkman A, Wolday D. Delayed clearance of Plasmodium falciparum in patients with human immunodeficiency virus co-infection treated with artemisinin. Ethiop Med J 2002; 40 Suppl 1:17-26.

(26) German P, Parikh S, Lawrence J et al. Lopinavir/ritonavir affects pharmacokinetic exposure of artemether/lumefantrine in HIV-uninfected healthy volunteers. J Acquir Immune Defic Syndr 2009; 51(4):424-429.

(27) World Health Organization. Guidelines for the treatment of malaria. 2006. WHO Press.

(28) Kublin JG, Patnaik P, Jere CS et al. Effect of Plasmodium falciparum malaria on concentration of HIV-1-RNA in the blood of adults in rural Malawi: a prospective cohort study. Lancet 2005; 365(9455):233-240.

(29) Pisell TL, Hoffman IF, Jere CS et al. Immune activation and induction of HIV-1 replication within CD14 macrophages during acute Plasmodium falciparum malaria coinfection. AIDS 2002; 16(11):1503-1509.

(30) Xiao L, Owen SM, Rudolph DL, Lal RB, Lal AA. Plasmodium falciparum antigen-induced human immunodeficiency virus type 1 replication is mediated through induction of tumor necrosis factor-alpha. J Infect Dis 1998; 177(2):437-445.

(31) Kaptein SJ, Efferth T, Leis M et al. The anti-malaria drug artesunate inhibits replication of cytomegalovirus in vitro and in vivo. Antiviral Res 2006; 69(2):60-69.

(32) Efferth T, Marschall M, Wang X et al. Antiviral activity of artesunate towards wild-type, recombinant, and ganciclovir-resistant human cytomegaloviruses. J Mol Med 2002; 80(4):233-242.

(33) Paeshuyse J, Coelmont L, Vliegen I et al. Hemin potentiates the anti-hepatitis C virus activity of the antimalarial drug artemisinin. Biochem Biophys Res Commun 2006; 348(1):139-144.

(34) Romero MR, Efferth T, Serrano MA et al. Effect of artemisinin/artesunate as inhibitors of hepatitis B virus production in an "in vitro" replicative system. Antiviral Res 2005; 68(2):75-83.

(35) Naesens L, Bonnafous P, Agut H, De CE. Antiviral activity of diverse classes of broad-acting agents and natural compounds in HHV-6-infected lymphoblasts. J Clin Virol 2006; 37 Suppl 1:S69-S75.

(36) Greenhouse B, Myrick A, Dokomajilar C et al. Validation of microsatellite markers for use in genotyping polyclonal Plasmodium falciparum infections. Am J Trop Med Hyg 2006; 75(5):836-842.

APPENDIX I
SCHEDULE OF EVALUATIONS

	Scenario #1: Separate Screening and Entry Visits		OR	Scenario #2: Combined Screening and Entry Visits										
	Screening [1, 2]	Study Day 0 Entry[2,3]		Screening/ Study Day 0 Entry [1, 2,3]	Study Day 1[2]	Study Day 2[2]	Study Day 3	Study Day 4	Study Day 8 (+24 hour window)[4]	Study Day 14 (+24 hour window)[4]	Study Day 28	Study Day 42 End of study	Unscheduled Sick Visit	Early Discontinuation[20]
CLINICAL EVALUATIONS														
Informed Consent	X			X										
History[5]	X	X		X	X	X	X		X	X	X	X	X	X
Physical exam[6]	X	X		X	X	X	X		X	X	X	X	X	X
Adherence Assessment[7]	X	X		X	X	X	X							
Artemether-lumefantrine (AL) dosing[8]		Dose 1- clinic Dose 2- home		Dose 1- clinic Dose 2- home	Dose 3- clinic	Dose 4- clinic Dose 5-home	Dose 6- clinic[9]							
LABORATORY EVALUATIONS														
Hematology[10]	1 mL			1 mL						1 mL	1 mL	1 mL		1 mL
Chemistries[11]	1 mL			1 mL						1 mL	1 mL	1 mL		1 mL
Thick Blood Smear (finger stick)	0.05	0.05		0.05	0.05	0.05	0.05		0.05	0.05	0.05	0.05	0.05	0.05
Thin Blood Smear (finger stick)	0.05			0.05										
Dried Blood Spots (DBS) for Malaria Infection[12]								0.1 mL	0.1 mL	0.1 mL	0.1 mL			
Urine [or Blood] Pregnancy test [13]	2 mL [1-2 mL] [17]	2 mL[16] [1-2 mL] [17]		2 mL [1-2 mL] [17]										
Virology														
DBS for Viral Load[14]	0.25 mL	0.25 mL[16]		0.25 mL					0.25 mL	0.25 mL		0.25 mL		0.25 mL
Drug Resistance Testing (DBS)[15]	0.25 mL	0.25 mL[16]		0.25 mL								0.25 mL		0.25 mL
Pharmacology														
Artemether-lumefantrine (AL) PK[18]							15 mL	3 mL	3 mL					
Nevirapine PK[19]		1 mL		1 mL			1 mL							
TOTAL BLOOD VOLUMES	2.6-4.6 mL	1.05-3.55 mL		3.6-5.6 mL	0.05 mL	0.05 mL	16.05 mL	3–3.1mL	3.3-3.4 mL	2.3-2.4 mL	2.05-2.15 mL	2.55 mL	0.05 mL	2.55 mL

APPENDIX I (Cont.)

1. Screening evaluations must be performed within 30 days of Entry.
2. Note that screening and entry can occur on the same day. In addition, for subjects who have started AL prior to study consent and have received up to 3 doses of AL, study Days 0, 1 and 2 evaluations may all be collapsed into a single visit, consistent with all criteria listed on study Day 0 (entry visit). Subjects who enter the study after having initiated AL may have a study Day 0 visit, with the next visit matched to the subject's day of AL treatment.
3. Subjects with Grade 3 or 4 hemoglobin at entry are allowed to participate in all study procedures (e.g. evaluation of the 28 and 42-day safety and toxicity) EXCEPT for the AL PK sampling or single samples for NVP measurements (to mimimize the volume of blood drawn in these children).
4. Team should be notified within 24 hours if timelines (i.e. +24 window) can not be met.
5. A complete history is required at Screening/Entry Visit; a targeted history [i.e. review of new illnesses and/or symptoms] is sufficient at subsequent visits.
6. Complete physical exam (including height, weight, vital signs [temperature, pulse, respiration, and blood pressure]) at Screening/Entry Visit and targeted physical exam (including vital signs [temperature, pulse, respiration, and blood pressure]) is sufficient at all other visits.
7. Adherence assessment will be conducted both retrospectively for the doses AL already taken, as well as, prospectively. All doses require documentation of date and time.
8. Dosing of AL must be performed according to schedule included in Section 5.1.
9. Draw Pre-Dose PK sample, then administer Dose 6 (the last dose) of AL.
10. Hematology should include: CBC.
11. Chemistries should include: AST, ALT, and serum creatinine.
12. These evaluations are only for subjects who, during study follow-up, experience repeat episodes of malaria infection (e.g. new infection or recrudescent infection). See section 6.214. Prepare 1 card with 2 spots at 0.05 mL each spot. Batched and shipped according to the instructions provided in the Laboratory Processing Chart (LPC). Note: DBS blood can be collected in a vacutainer tube ONLY when blood is collected for other laboratory evaluations (i.e. hematology, chemistry, or pharmacokinetic). All other DBS collections should be performed via finger stick. See Appendix III for additional collection, processing, and storage instructions.
13. Must be performed within 72 hours of enrollment on female subjects who have had their first menstrual period.
14. Prepare 1 card with 5 spots at 0.05 mL each spot. Batched and must be performed at DAIDS VQA-certified laboratory. Note: DBS blood can be collected in a vacutainer tube ONLY when blood is collected for other laboratory evaluations (i.e. hematology, chemistry, or pharmacokinetic). All other DBS collections should be performed via finger stick. See Appendix III for additional collection, processing, and storage instructions.
15. Prepare 1 card with 5 spots at 0.05 mL each spot. Collect on all study participants. To be performed on subjects who show changes in viral load pattern between study Day 0 and study Day 42 using dried blood spot collected at screening and study Day 42. Note: DBS blood can be collected in a vacutainer tube ONLY when blood is collected for other laboratory evaluations (i.e. hematology, chemistry, or pharmacokinetic). All other DBS collections should be performed via finger stick. See Appendix III for additional collection, processing, and storage instructions.
16. This evaluation should only be performed if it was not performed at Screening.
17. Blood pregnancy test can be substituted for urine pregnancy test.
18. AL pharmacokinetics (3 mL per sample) will be collected on study Day 3: Pre-dose, 1, 2, 4, and 8 hours; study Day 4: 24 hours; and study Day 8: 120 hours post-administration of the last AL dose. Participants will be asked to remain within the clinic or research facility until completion of the 8 hour blood sample collection at which time they will be discharged for home. They will be asked to return to the clinic or research facility for the 24 hour (study Day 4) and 120 hour (study Day 8) blood sample collections. For subjects who experience treatment failure before study Day 3, PK sampling will be discontinued. Specimen aliquots are prepared according to instructions provided in the LPC.
19. NVP pharmacokinetic samples will be collected on study Day 0 and study Day 3. The baseline (study Day 0) pharmacokinetic samples of NVP will be collected at random times prior to initiating AL treatment and following the previous NVP dose (approximately 12 hours following the evening dose of NVP). The second sample, study Day 3, will be collected just prior to the morning NVP dose and at the time of the last dose of AL. Specimen aliquots are prepared according to instructions provided in the LPC.
20. Subjects who prematurely discontinue study treatment will continue to be followed at scheduled study visits, but only the evaluations listed in this column are required.

For insufficient blood draws at SCREENING visit, priorities are as follows:

a. Hematology
b. Chemistries
c. Nevirapine PK

For insufficient blood draws at DAY 3 visit, priorities are as follows:

a. Artemether-lumefantrine (AL) PK
b. Nevirapine PK

Appendix II

BLOOD VOLUME COLLECTION IN SMALL CHILDREN

The total amount of blood collected per subject during the trial will be about 35 mL of which up to 21 mL will be collected in the first 72 hours. Recommendations as to the acceptable amount of blood to be drawn from subjects specifically for research purposes are variable and largely depend on individual institutions. The most conservative recommendations suggest no more than 2.5% (~2ml/kg) of total blood volume within a 24 hour period or 5% (~4mL/kg) within a 30 day period. The National Institutes of Health (NIH) guidelines state that no more than 3mL/kg are to be drawn in a single blood draw (or 24 hour period) and no more than 9.5mL/kg are to be drawn over any eight week period for the purposes of research in children.

Example: A 3 year old girl weighing 12kg (15% weight-for-age) has an approximate blood volume of 840mL (70mL/kg total blood volume x 12kg). An 21 mL blood draw represents approximately 2.5% of total blood volume for this participant. Alternatively, this represents 1.75mL/kg volume of blood drawn in a 24 hour period, well below the 3mL/kg recommended by the NIH.

In the case where a seemingly eligible participant has had blood drawn, or will have blood drawn for any reason, such that the total volume blood being drawn over any 8 week period will exceed 9.5 mL/kg, the subject will be considered ineligible for this PK study.

APPENDIX III
DRIED BLOOD SPOT COLLECTION, PROCESSING AND STORAGE INSTRUCTIONS

I. General Instructions

The Dried Blood Spot (DBS) cards should be collected on Whatman Protein Saver Cards (Whatman 10534612). DBS cards should be prepared from blood drawn in EDTA (purple top) tubes.

The filter cards come with printed circles. Always apply blood to the inside of the circles. Fill the entire circle with blood (50 microliters). Apply blood to only one side of the filter paper (the side with the printing).

Avoid using capillary tubes to collect blood specimens. There exists considerable danger of infection for lab workers from puncture wounds resulting from the accidental breakage of the capillary tubes.

Figure 1. Dried Blood Spot Card

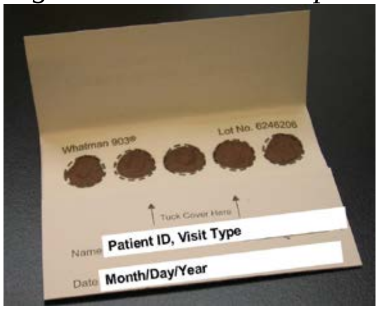

II. Biosafety

It is essential that universal precautions be taken while working with these specimens. Dried blood spots on filter paper are not considered to be a biohazard. However, you may want to double glove and wear a lab coat/gown at all times even with DBS to ensure safe handling of samples. If you should tear a glove, remove the torn one and replace it immediately. If a needle puncture should occur, notify the lab manager or project coordinator immediately and appropriate action will take place.

III. Preparation of DBS Using Fingerprick Specimen

Equipment Needed:

1. Unistick 2 device (depth of 1.8 mm)
2. Alcohol swab

3. Dry sterile gauze pad
4. Whatman Protein Saver Card (Whatman 10534612)
5. Gas impermeable storage bag
6. Indicator desiccant pack (such as Whatman WB100003)

Procedure:

Label DBS card with PID/SID, date, time of day drawn, and study day. Position the patient. The patient should sit in a chair, lie down or sit up in bed. Hyperextend the patient's arm. Cleanse the 3rd or 4th finger with alcohol and dry with sterile gauze.

(Do not use the tip of the finger or the center of the finger. Avoid the side of the finger where there is less soft tissue, where vessels and nerves are located, and where the bone is closer to the surface. The 2nd (index) finger tends to have thicker, callused skin. The fifth finger tends to have less soft tissue overlying the bone. Avoid puncturing a finger that is cold or cyanotic, swollen, scarred, or covered with a rash.)

Clean the area of the finger with an alcohol pad and allow the area to air dry. Prepare the Unistick 2 device by depressing the pink plunger until it clicks. Twist the plunger until it breaks off and remove it from the device. The Unistick 2 device is now ready for use. Do not touch the cleaned area or allow the finger to come into contact with any non-sterile item or surface.

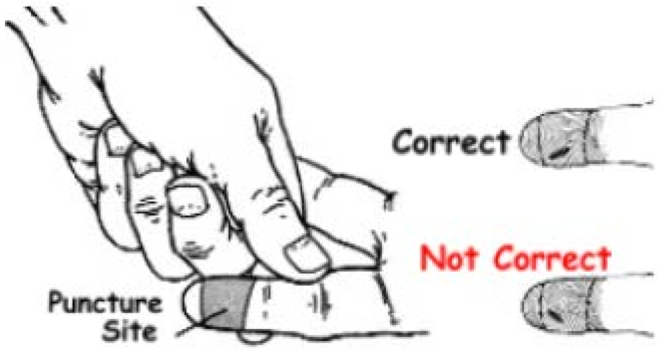

Wipe away the first drop of blood using a dry sterile gauze pad. The blood is then allowed to flow to be collected on the dried blood spot card. To do this, gently touch the filter paper card to the blood drop. Allow the card to absorb the blood until the circle is <u>FULL</u>. You should be able to obtain 5 spots of blood on a card. You may need to squeeze the finger to obtain more blood; however, do not milk the finger as it mixes interstitial fluid with the blood. Once all 5 spots are obtained, gently press a sterile pad to the site until bleeding has stopped. The finger must then be monitored for bleeding. Placing a sterile pad will help prevent a hematoma from forming.

IV. Preparation of DBS using Venous blood collected in EDTA tube

Equipment Needed:

1. Vacutainer Evacuated Blood Collection Tubes – these tubes are designed to be filled with a predetermined volume of blood by vacuum. The rubber stoppers are color-coded according to the additive that the tube contains. Collect 1 EDTA (purple top) tube at each time point required. Total volume required per card will be 250 microliters to fill five spots each at 50 microliters per spot.
2. Alcohol swab
3. Tourniquet
4. Bandage/Plaster
5. Vacutainer Needle
6. Vacutainer Needle Holder
7. Protein Saver Card (Whatman 10534612)
8. Gas impermeable storage bag
9. Indicator desiccant pack (such as Whatman WB100003)

Procedure:

Standard procedures for labeling should be followed. The tubes should be labeled with the patient ID number, visit timepoint, and the date of collection. When recording date of collection, be sure to distinguish in your notes to the processing laboratory what orientation you are recording (Month/Day/Year or Day/Month/Year). Fill the blood collection tube to the recommended volume so the anticoagulant is at the proper dilution. Gently invert the tube (5-10 times) to mix thoroughly. After the blood is completely mixed, remove the cap, and take 50 microliters of the whole blood and apply to a single spot. Repeat four additional times to fill all five spots on the card. This transfer of blood should be performed with a pipette and a disposable tip. If a pipette with a disposable tip is unavailable, the team suggests using a disposable polyethylene pipette pictured below.

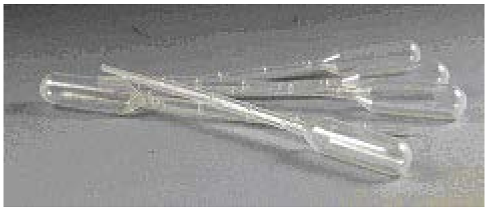

V. Handling and Storage of Dried Blood Spots

Care should be taken to not touch the DBS circle once blood is collected. Allow the blood spot to air dry in a clean, dry place overnight (protected from rodents and insects). The following day, place the DBS card in a gas impermeable bag with an indicator

dessicant pack (see supplies suggestion). Store no more than one card per bag. Once in the bag, DBS should be stored at -20 degrees Celsius until shipment to central laboratory. (For collection sites where -20 degrees Celsius storage is unavailable, the DBS card may be stored at room temperature for a maximum of two weeks before shipment to central laboratory).

DBS cards should be stored at -20 degrees Celsius at central laboratory until shipment to processing laboratory. DBS cards can be shipped at ambient temperature to processing laboratory. Once received at processing laboratory, samples should be then stored at -20 degrees Celsius.

Figure 2. Storage of DBS

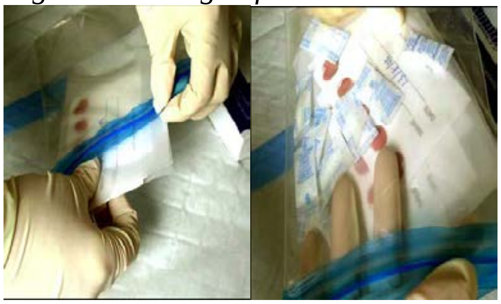

VI. Shipment of Dried Blood Spots

All of the individual dried blood spot cards in their corresponding gas impermeable storage bag with indicating dessicant are to be shipped together within a manila envelope. The whole blood is deemed non-infectious once dried on the filter cards; hence DO NOT USE a Biological Substance, Category B sticker for shipment.

The processing laboratory's address is:

Dr. Deborah Persaud's Laboratory
Johns Hopkins University
720 Rutland Avenue
Ross Building Room 1171
Baltimore, MD 21205 USA
Phone: (443) 287-3733
Fax: (410) 614-1315

The processing laboratory address for DNA-Fingerprinting of Parasites-ONLY is:

Sunil Parikh, M.D., M.P.H.
University of California – San Francisco
San Francisco General Hospital
1001 Potrero Avenue, Building 30, Room 3402
San Francisco, CA 94110

APPENDIX IV

GUIDANCE FOR DETERMINING SEVERE MALNUTRITION

The below information are referenced from the World Health Organization (WHO) [http://www.who.int/en/] website.

1. Body Mass Index (BMI) is calculated as (Weight, in kilograms)^2 / (Height, in meters)

2. Table for determining if BMI Z-score < -3SD in BOYS ≥5 years old: http://www.who.int/growthref/bmifa_boys_5_19years_z.pdf

3. Table for determining if BMI Z-score < -3SD in GIRLS ≥5 years old: http://www.who.int/growthref/bmifa_girls_5_19years_z.pdf

4. Table for determining if Weight-for-Height < -3SD in BOYS <5 years old: See Appendix I, pp 41-42 at http://www.who.int/nutrition/publications/severemalnutrition/en/manage_severe_malnutrition_eng.pdf

5. Table for determining if Weight-for-Height < -3SD in GIRLS <5 years old: See Appendix I, pp 41-42 at http://www.who.int/nutrition/publications/severemalnutrition/en/manage_severe_malnutrition_eng.pdf

APPENDIX V

COARTEM® DISPERSIBLE DOSAGE AND ADMINISTRATION DIAGRAM

Dosage and Administration: Dispersible tablets for oral administration. The dispersible tablet(s) composing 1 dose should be completely dispersed in a small amount of water (approximately 10 mL per tablet). Stir gently and administer immediately to the subject. Rinse the glass with an additional small amount of water (approximately 10 mL) and give immediately to the subject.

Although subjects with acute malaria are frequently intolerant of food, AL should be followed by fluids (particularly drinks containing fats, such as milk) and if possible, with a normal diet as soon as the subject can tolerate food. Ingestion with fat-containing food and drinks massively improves absorption.

For this study, every dose of AL must be administered with whole milk. Although any risk of recrudescence is very small, subjects who remain averse to food during treatment should be closely monitored.

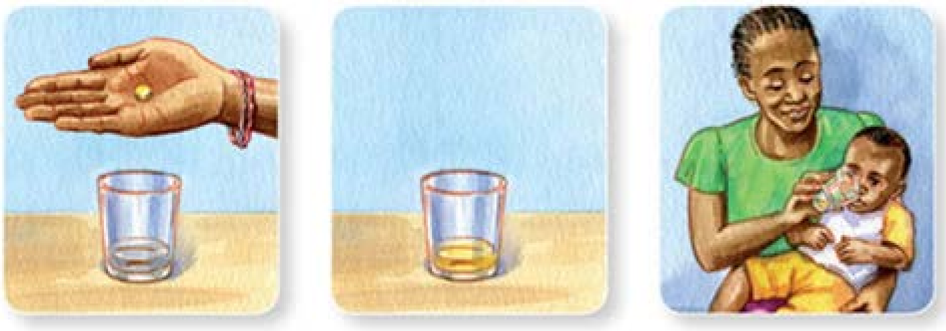

APPENDIX VI

IMPAACT SAMPLE INFORMED CONSENT TEMPLATE

DIVISION OF AIDS
INTERNATIONAL MATERNAL PEDIATRIC ADOLESCENT AIDS CLINICAL TRIALS GROUP (IMPAACT)

SAMPLE INFORMED CONSENT

For protocol:

PHARMACOLOGY OF ARTEMISININ-BASED ANTIMALARIAL THERAPY WITHIN THE CONTEXT OF ANTIRETROVIRAL THERAPY, P1079, DRAFT
Version 1.0, dated May 21, 2010

SHORT TITLE FOR THE STUDY: PHARMACOLOGY OF ARTEMISININ-BASED ANTIMALARIAL THERAPY, Version 1.0, dated May 21, 2010

INTRODUCTION

Your child is being asked to take part in this research study because your child is infected with HIV, the virus which causes Acquired Immune Deficiency Syndrome (AIDS) and is also infected with malaria, requiring treatment with Coartem® Dispersible (a trademark brand of the anti-malaria medicine). Malaria is a disease caused by a parasite and is spread by mosquito bites. This study is sponsored by the United States National Institutes of Health (NIH). The doctor in charge of this study at this site is: (insert name of Principal Investigator). Before you decide if you want your child to be a part of this study, we want you to understand the study.

This is a consent form. It gives you information about this study. The study staff will talk with you about this information. You are free to ask questions about this study at any time. If you agree to allow your child to take part in this study, you will be asked to sign this consent form. You will get a copy to keep.

WHY IS THIS STUDY BEING DONE?

The purpose of this study is to see if taking nevirapine (NVP) for HIV changes the way Coartem® Dispersible works in children who are co-infected with both HIV and malaria. This study will compare the blood levels of Coartem® Dispersible in co-infected children who take NVP with the co-infected children who do not take anti HIV medicines. The

study will also assess the safety of using both medications, Coartem® Dispersible and NVP in children. NVP has been approved for use in children with HIV by the US Food and Drug Administration (FDA). Coartem® Dispersible has also been approved for use in children by the FDA.

WHAT DOES MY CHILD HAVE TO DO IF HE/SHE IS IN THIS STUDY?

Your child will have at least 9 visits during the 6 weeks that he/she will be taking part in this study, as indicated on the P1079 Subject Visit Schedule below.

(Note to Sites: you may choose to use this supplemental tool if you think it will be helpful when obtaining consent)

	Study Day 0 (Screening & Entry)	Study Day 1	Study Day 2	Study Day 3	Study Day 4	Study Day 8	Study Day 14	Study Day 28	Study Day 42 End of study	Unscheduled Sick Visit	Early Discontinuation
Informed Consent	X										
History	X	X	X	X		X	X	X	X	X	X
Physical exam	X	X	X	X		X	X	X	X	X	X
Coartem® Dispersible given at Clinic	X	X	X	X							
Coartem® Dispersible given at home*	X		X								
Finger Prick to test for malarial infection	X	X	X	X		X	X	X	X	X	X
Blood Collected	X			X	X	X	X	X	X		X
Pregnancy test**	X										

* A dosing schedule will be provided to you at the clinic so that you can record the dose and time that you give your child his/her medication. The study staff will ask you for this information so that they can keep track of your child's anti-malaria treatment.

**Pregnancy test will be performed only on girls who have had their first menstrual period.

Before starting this study ("Screening Visit") [*sites: add local information regarding how long study visits will take*].
After you have read and signed this consent form, your child will have the following done at the screening visit to see if your child can participate in the study:

- You will be asked questions about your child's medications (anti-HIV and anti-malarial) and medical history. You may be asked for permission to review your child's medical record.
- Your child will have a complete physical exam including height, weight, and vital signs (temperature, blood pressure, pulse, and respiratory rate).
- If your child is female and has had her first menstrual period, your child will provide a urine or blood sample for a pregnancy test. The test must show that your child is not pregnant in order for your child to participate in this study.
- Your child will have about ½ teaspoon (2.5 mL) of blood drawn:
    - for routine blood tests,
    - to test for malarial infection (finger prick for a small blood sample to have the laboratory check for the amount of malarial parasites in your child's blood,
    - to measure your child's HIV viral load (how much HIV is in your child's blood), and
    - to test for HIV resistance to HIV drugs (changes in your child's HIV)

The results of these tests will determine if your child is eligible to enter the study.

If your child has already started Coartem® Dispersible, your child will be eligible to enter the study if the:

- total number of doses prior to entry does not exceed 3 doses, and
- drug and dose your child has been receiving is the same drug and dose your child will take as part of this study.

Throughout the study, you will be told the results of the malaria tests, viral load and routine blood tests which are being done for the study.

During the Study [*Sites: add local information regarding how long study visits will take*].

Entry Visit (Study Day 0) (this visit may occur on the same day as the screening visit)
Once it is known that your child can participate in this study, your child will have an entry visit. At this visit the following will be done:

- You will be asked questions about your child's medical history.
- Your child will have a complete physical exam.
- Your child will have about ⅓ teaspoon (1.5 mL) of blood drawn:
    - to measure your child's HIV viral load (how much HIV is in your child's blood),

  - ➢ to test for HIV resistance to HIV drugs,
  - ➢ to find out how much NVP is in your child's body
- Your child will have his/her finger pricked to check for the amount of malarial parasites in your child's blood.

- Your child will be treated for malaria with a tablet called Coartem® Dispersible which will be put in a small amount of water to dissolve. You will be given instructions on how to dissolve the Coartem® Dispersible tablet and how to give it to your child. Each time your child is given a dose of the anti-malaria medicine, he/she will also be given a glass of whole milk to drink. The milk helps to increase the levels of the medicine in the blood. Your child will receive one dose of Coartem® Dispersible at the clinic, between 11:00 AM and 2:00 PM, OR between 2:00 PM and 5:00 PM. Depending on when the first dose of Coartem® Dispersible is given, your child will receive another dose again at home in the evening, between 9:00 PM and 12:00 AM, OR between 10:00 PM and 1:00 AM. You must record the time and dose that you gave your child his/her medication. A dosing schedule will be provided to you at the clinic so that you can record the dose and time that you give your child his/her medication. The study staff will ask you for this information so that they can keep track of your child's anti-malaria treatment.

<u>Study Day 1:</u>
At this visit the following will be done:
- You will be asked questions about your child's symptoms, medications and medical history since the last visit.
- Your child will have a short physical exam to assess any symptoms which are present.
- Your child will have his/her finger pricked to check for the amount of malarial parasites in your child's blood.
- Your child will receive one dose of Coartem® Dispersible at the clinic, between 1:00 PM and 3:00 PM.

This visit will take about 30 minutes.

<u>Study Day 2:</u>
At this visit the following will be done:

- You will be asked questions about your child's symptoms, medications and medical history since the last visit.
- Your child will have a short physical exam to assess any symptoms which are present.
- Your child will have his/her finger pricked to check for the amount of malarial parasites in your child's blood.
- Your child will receive one dose of Coartem® Dispersible at the clinic, between 8:00 AM and 10:00 AM and another dose again at home in the evening, between 8:00 PM and 10:00 PM. You must record the time and dose that you gave your child his/her medication.

This visit will take about 30 minutes.

<u>Study Day 3:</u>
At this visit the following will be done:

- You will be asked questions about your child's symptoms, medications and medical history since the last visit.
- Your child will have a short physical exam to assess any symptoms which are present.
- Your child will have his/her finger pricked to check for the amount of malarial parasites in your child's blood.
- Your child will receive the last dose of Coartem® Dispersible at the clinic, between 8:00 AM and 10:00 AM.
- Your child will have about 3 teaspoons (16 mL) of blood drawn:
    - ➢ to measure the amounts of Coartem® Dispersible in your child's blood over time. Blood will be drawn 5 times over 8 hours (before the medicine, and 1, 2, 4, and 8 hours after the medicine). Your child will need to stay in the clinic for the full 8 hours during this testing.
    - ➢ to find out how much NVP is in your child's blood.

This visit will take about 8 ½ hours.

<u>Study Day 4:</u>
At this visit the following will be done:

- Your child will have about ½ teaspoon (3 mL) of blood drawn:
    - ➢ to measure the amount of Coartem® Dispersible in your child's blood.
- If your child experiences a repeat malaria infection (i.e. new or old infection), your child will have his/her finger pricked to check for the amount of malarial parasites in your child's blood.

This visit will take about 30 minutes.

Study Day 8:
At this visit the following will be done:

- You will be asked questions about your child's symptoms, medications and medical history since the last visit.
- Your child will have a short physical exam to assess any symptoms which are present.
- Your child will have his/her finger pricked to check for the amount of malarial parasites in your child's blood.
- Your child will have about ½ teaspoon (3 mL) of blood drawn:
    - to measure your child's HIV viral load, and
    - to measure the amount of Coartem® Dispersible in your child's blood.

This visit will take about 30 minutes.

Study Days 14, 28, and 42:
At these visits the following will be done:

- You will be asked questions about your child's symptoms, medications and medical history since the last visit.
- Your child will have a short physical exam to assess any symptoms which are present.
- Your child will have his/her finger pricked to check for the amount of malarial parasites in your child's blood.
- Your child will have about ½ teaspoon (2 – 2.5 mL) of blood drawn:
    - for routine blood tests,
    - to measure your child's HIV viral load (study Days 14 and 42 only), and
    - to test for HIV resistance (study Day 42 only).

These visits will take about 30 minutes each.

Sick visit(s):
If your child becomes sick during the study, he/she will need to return to the clinic for the following:

- You will be asked questions about your child's symptoms and medical history.
- Your child will have a short physical exam.
- Your child will have his/her finger pricked to have the laboratory check for the amount of malarial parasites in your child's blood.

Early discontinuation from the study:
If your child discontinues the study before the Day 42 study visit, he/she will need to return to the clinic for the following:

- You will be asked questions about your child's symptoms and medical history.
- Your child will have a short physical exam.
- Your child will have his/her finger pricked to have the laboratory check for the amount of malarial parasites in your child's blood.
- Your child will have about ½ teaspoon (2.5 mL) of blood drawn:
    - for routine blood tests,
    - to measure your child's HIV viral load, and
    - to test for HIV resistance.

OTHER INFORMATION

- If your child becomes pregnant during the 42 days study follow-up period, your child will be referred to another clinic for follow-up.

- Sometimes a heparin lock (small plastic tube) is used when collecting more than 1 blood sample over a period of time. It is left in the vein until all of the blood draws are completed, and then it is removed. This allows blood to be taken repeatedly without having to stick your child with a needle many times.

- The information collected in this study may be used for other IMPAACT-approved HIV-related research.

HOW MANY PEOPLE WILL TAKE PART IN THIS STUDY?

About 48 children will take part in this study; 24 children who are taking nevirapine, and 24 children who are not taking nevirapine.

HOW LONG WILL MY CHILD BE IN THIS STUDY?

Your child will be in this study for about 42 days.

WHY WOULD THE DOCTOR TAKE MY CHILD OFF THIS STUDY EARLY?

The study doctor may need to take your child off the study early without your permission if:

- The study is stopped or cancelled by the National Institutes of Health (NIH), the Office for Human Research Protections (OHRP), other country-specific government agencies, IMPAACT Network, Ethics Committee (EC), or the site's Institutional Review Board (IRB). An IRB is a committee that watches over the safety and rights of research subjects.
- Your child is not able to attend the study visits as required by the study.

- Your child needs to begin antiretroviral treatment while enrolled in the study. If your child needs to begin antiretroviral treatment during the study, your child will not be followed for their HIV care through P1079.
- The investigator determines that further participation would be detrimental to your child's health or well-being.

WHAT ARE THE RISKS OF THE STUDY?

Risks When Drawing Blood or Using a Heparin Lock:

Your child may feel some discomfort when blood is drawn for this study. Other risks may include bleeding, bruising and swelling or a small blood clot may form where the needle enters the skin. There is also a small risk of infection from drawing blood.

Coartem® Dispersible Risks:

After taking the anti-malaria medicine, your child may develop a rash or skin irritation. Other possible (but unlikely) side effects may include nausea and vomiting, generally within 1 hour of the first dose, abdominal pain, loss of appetite, diarrhea, temporary loss of hearing (low risk), and/or low red blood count.

ARE THERE RISKS RELATED TO PREGNANCY?

Coartem® Dispersible is the drug of choice for pregnant women who develop malaria. The drug is important for treatment as it may reduce the risk for developing placental malaria that is common in pregnant women. The drug is considered to be safe in pregnancy.

If your child is female, has had her first menstrual period, and having sex that could lead to pregnancy, your child must agree not to become pregnant. If your child can become pregnant, your child must use at least two methods of reliable birth control that you discuss with the study staff throughout the whole study.

If you/your child think(s) you/they may be pregnant at any time during the study, tell your study staff right away. If your child is found to be pregnant after entering the study, your child will be allowed to continue participating in the study.

ARE THERE BENEFITS TO TAKING PART IN THIS STUDY?

If your child takes part in this study, there may be a direct benefit to your child, because your child's blood will be checked during the study to find out if the malaria clears from your child's body after taking the Coartem® Dispersible, but no guarantee can be made.

It is also possible that your child may receive no benefit from being in this study. Information learned from this study may help others who have HIV.

WHAT OTHER CHOICES DOES MY CHILD HAVE BESIDES THIS STUDY?

Instead of being in this study you have the choice of:
- treatment with other prescription drugs available to your child
- treatment with experimental drugs, if your child qualifies
- not participating in the study

Please talk to your doctor about these and choices available to your child. Your doctor will explain the risks and benefits of these choices.

WHAT ABOUT CONFIDENTIALITY?

Every effort will be made to keep your child's personal information confidential. We cannot guarantee absolute confidentiality. Your child's personal information may be disclosed if required by law. Any publication of this study will not use your child's name or identify your child personally.

Your child's records may be reviewed by the Office for Human Research Protections (OHRP), (insert name of site) IRB, Ethics Committee (EC), the National Institute of Health (NIH), study staff, study monitors, local and national regulatory authorities.

WHAT ARE THE COSTS TO ME?

The study will provide Coartem® Dispersible at no cost to you. Taking part in this study may lead to added costs to you and your insurance company. In some cases it is possible that your insurance company will not pay for these costs because your child is taking part in a research study.

WILL I RECEIVE ANY PAYMENT?

No, you will not receive payment for your child's participation in this study.

WHAT HAPPENS IF MY CHILD IS INJURED?

If your child is injured as a result of being in this study, your child will be given immediate treatment for his/her injuries. The cost for this treatment will be charged to you or your insurance company. There is no program for compensation either through this institution or the National Institutes of Health (NIH). You will not be giving up any of your legal rights by signing this consent form.

WHAT ARE MY CHILD'S RIGHTS AS A RESEARCH SUBJECT?

Taking part in this study is completely voluntary. You may choose not to allow your child to take part in this study or take your child out of the study at any time. Your decision will not result in any penalty or loss of benefits to which you are otherwise entitled.

The study doctor will tell you about new information from this or other studies that may affect your child's health, welfare or willingness to stay in this study. If you want the results of the study, you should tell the study staff.

WHAT DO I DO IF I HAVE QUESTIONS OR PROBLEMS?

For questions about this study or a research-related injury, contact:

- name of the investigator or other study staff
- telephone number of above

For questions about your child's rights as a research subject, contact:

- name or title of person on the Institutional Review Board (IRB) or other organization appropriate for the site
- telephone number of above

SIGNATURE PAGE

If you have read this consent form (or had it explained to you), all your questions have been answered and you agree to take part in this study, please sign your name below.

______________________	______________________
Participant's Name (print)	Participant's Signature and Date
______________________	______________________
Participant's Legal Guardian (print)<br>(As appropriate)	Legal Guardian's Signature and Date
______________________	______________________
Study Staff Conducting<br>Consent Discussion (print)	Study Staff Signature and Date
______________________	______________________
Witness' Name (print)<br>(As appropriate)	Witness's Signature and Date
