# Supplementary material for: Concomitant nevirapine impacts pharmacokinetic exposure to the antimalarial artemether-lumefantrine in African children
Source: PLoS One. 2017 Oct 24;12(10):e0186589. doi: 10.1371/journal.pone.0186589 (PMC5655345; doi:10.1371/journal.pone.0186589)
Supplement: S2 Table — (RTF) [file pone.0186589.s004.rtf]

S2 Table: Characteristics of P1079 participants at study entry

	Prior ARVs		
Characteristic		NVP
(N=16)	No ARVs
(N=3)	Total
(N=19)	
Race	Black African	16 (100%)	3 (100%)	19 (100%)	
Age (yrs)	N	16	3	19	
	Mean (s.d.)	7.4 (2.1)	7.3 (3.3)	7.4 (2.2)	
	<6 yrs	4 (25%)	1 (33%)	5 (26%)	
	>=6 yrs	12 (75%)	2 (67%)	14 (74%)	
WHO clinical disease stage	Clinical stage I	2 (13%)	3 (100%)	5 (26%)	
	Clinical stage II	4 (25%)	0 (0%)	4 (21%)	
	Clinical stage III	8 (50%)	0 (0%)	8 (42%)	
	Clinical stage IV	2 (13%)	0 (0%)	2 (11%)	
On TMP/SMX	No	2 (13%)	1 (33%)	3 (16%)	
	Yes	14 (88%)	2 (67%)	16 (84%)	
Weight (kg)	N	16	3	19	
	Mean (s.d.)	20.0 (4.6)	20.9 (5.6)	20.1 (4.6)	
WHO Weight-for-age z-score (0-10 yrs)	N	13	3	16	
	Mean (s.d.)	-1.45 (0.96)	-0.87 (2.00)	-1.34 (1.15)	
	Min, Max	-2.64, 0.29	-3.17, 0.39	-3.17, 0.39	
	Median	-1.95	0.18	-1.57	
Height(cm)	N	16	3	19	
	Mean (s.d.)	113.2 (12.5)	108.1 (18.2)	112.4 (13.1)	
	Min, Max	90, 140	90.0, 126.4	90, 140	
	Median	112	108	112	
WHO Length/height-for-age z-score (0-19 yrs)	N	16	3	19	
	Mean (s.d.)	-1.93 (1.02)	-2.55 (2.16)	-2.03 (1.20)	
	Min, Max	-3.54, -0.44	-4.77, -0.46	-4.77, -0.44	
	Median	-1.84	-2.43	-1.85	
BMI calculation	N	16	3	19	
	Mean (s.d.)	15.4 (1.2)	17.9 (1.7)	15.8 (1.5)	
	Min, Max	13.9, 17.8	16.9, 19.9	13.9, 19.9	
	Median	15.3	16.9	15.6	
WHO BMI-for-age z-score (0-19 yrs)	N	16	3	19	
	Mean (s.d.)	-0.27 (0.87)	1.17 (1.37)	-0.04 (1.06)	
	Min, Max	-1.77, 1.30	0.14, 2.73	-1.77, 2.73	
	Median	-0.32	0.64	-0.02	
WHO weight-for-length/height z-score (0-5 yrs)	N	2	1	3	
	Mean (s.d.)	0.52 (0.85)	2.59 (.)	1.21 (1.34)	
	Min, Max	-0.08, 1.12	2.59, 2.59	-0.08, 2.59	
	Median	0.52	2.59	1.12	
